# Severity of clinical dry eye manifestations influences protein expression in tear fluid of patients with primary Sjögren’s syndrome

**DOI:** 10.1371/journal.pone.0205762

**Published:** 2018-10-12

**Authors:** Lara A. Aqrawi, Xiangjun Chen, Janicke Liaaen Jensen, Mathias Kaurstad Morthen, Bernd Thiede, Øygunn Aass Utheim, Øyvind Palm, Behzod Tashbayev, Tor Paaske Utheim, Hilde Kanli Galtung

**Affiliations:** 1 Department of Oral Surgery and Oral Medicine, Faculty of Dentistry, University of Oslo, Oslo, Norway; 2 The Norwegian Dry Eye Clinic, Oslo, Norway; 3 Department of Biosciences, University of Oslo, Oslo, Norway; 4 Department of Medical Biochemistry, Oslo University Hospital, Oslo, Norway; 5 Department of Rheumatology, Oslo University Hospital, Oslo, Norway; 6 Department of Plastic and Reconstructive Surgery, Oslo University Hospital, Oslo, Norway; 7 Department of Oral Biology, University of Oslo, Oslo, Norway; Universitat Regensburg, GERMANY

## Abstract

Ocular dryness is a characteristic feature of primary Sjögren’s syndrome (pSS). This may result in dry eye disease (DED), leading to damage of the ocular surface. Additional, non-invasive diagnostic techniques are needed when evaluating pSS patients. Hence, screening for disease-specific biomarkers in biological fluid could be promising. We have previously examined the proteome of tear fluid from pSS patients through Liquid chromatography-mass spectrometry (LC-MS), and conducted a thorough ocular evaluation of patients with pSS. In this study we further explored the association between dry eye manifestations and protein expression in tear fluid of pSS patients. Medical history of 27 patients and 32 healthy controls was gathered. Subjective complaints were registered through questionnaires. Objective findings including tear osmolarity, tear film break up time (TFBUT), Schirmer’s test, and ocular and corneal surface staining were also recorded. LC-MS was conducted formerly on tear fluid from all subjects in order to generate proteomic biomarker profiles. Scaffold was employed to analyse the LC-MS data for quantitative differences between patient and control groups, and the mean spectral counts were calculated for the five most upregulated proteins in relation to DED manifestations. Dysregulated cellular processes were identified in pSS patients using FunRichv3 enrichment analysis. The five most upregulated proteins previously identified in pSS patients were DNA (apurinic or apyrimidinic site) lyase (APEX1), thioredoxin-dependent peroxidase reductase (PRDX3), copine (CPNE1), aconitate hydratase (ACO2), and LIM domain only protein 7 (LMO7), in descending order. A significant increase in mean spectral counts for these proteins were observed in pSS patients with pathological DED manifestations compared to healthy controls (p<0.0001). Consequently, dysregulated cellular pathways involving innate and adaptive immunity were also detected. In conclusion, our observations suggest a relationship between presence of dry eye signs and upregulated proteins in tear fluid from patients with pSS. Further studies are needed in order to replicate the concepts explored and analyses performed in a greater cohort of pSS patients, where sensitivity and specificity of the methods conducted can also be verified further.

## Introduction

Sjögren’s syndrome (SS) is an autoimmune disorder, where chronic inflammation results in progressive destruction of connective tissue, primarily the lacrimal and salivary glands [1, 2]. It is divided into primary (pSS) when it develops independently, and secondary (sSS) when in combination with other rheumatic diseases, such as rheumatoid arthritis and systemic lupus erythematosus [3, 4]. More than 95% of the patients with pSS display symptoms of ocular and oral dryness (sicca symptoms), which are characteristic features of this disease [5]. In some instances, ocular dryness also results in dry eye disease (DED); a multifactorial disease of the tears and ocular surface that is characterised by tear film instability, according to the TFOS International Dry Eye Workshop II (DEWS). It is also associated with inflammation of the ocular surface and symptoms of ache and discomfort [6].

SS had been known to have a prevalence ranging between 0.01% and 0.6%, where 90% of those affected are female, mostly middle-aged [7–9]. For diagnosis of pSS patients, the American-European Consensus Group (AECG) criteria from 2002 are the main classification criteria used today [10], although new ACR classification criteria have also been introduced [11]. These criteria involve the evaluation of symptoms encompassing ocular and oral dryness, measuring the secretory ability of the exocrine glands (tear and saliva secretion), screening for disease-specific autoantibodies known as anti-Ro/SSA (anti-Ro60 and anti-Ro52) and anti-La/SSB, and evaluating minor salivary gland biopsies for mononuclear cell infiltration [12]. The routine assessment of minor salivary glands applies a semi-quantitative, invasive technique useful for classifying patients with glandular dysfunction that do not display peripheral autoantibody production [13]. Nonetheless, an unmet need for non-invasive, more accurate diagnostic tools still remains for pSS. Hence, exploring additional techniques, such as screening for disease-specific biomarkers, has been in focus in recent years [14, 15]. Such an approach could potentially also be used for disease staging and monitoring. Indeed, the application of liquid chromatography-mass spectrometry (LC-MS) in order to study the proteome of biological fluids has aided in identifying potential biomarkers and therapeutic targets in several rheumatic diseases, including pSS [16].

To further understand the pathogenesis of pSS, both saliva [15–21] and tear fluid [22, 23] have formerly been used to distinguish potential biomarkers for this disease. This provided new insight into molecular pathways that are dysregulated, comprising of either downregulated or upregulated genes that promote anti- and/or pro-inflammatory signalling, and successively inflammation [24]. Such altered pathways could in turn play a central role in both innate and adaptive immune responses of patients with pSS.

We have previously explored potential biomarkers in tear fluid and saliva of pSS patients (**S1 File**) [24], and also conducted a comprehensive and thorough ocular evaluation of patients with pSS (**S2 File**) [25]. Taking the aforementioned parameters into account, a thorough analysis of the relationship between recently suggested biomarkers in pSS, clinical dry eye manifestations, and how this in turn relates to alterations in innate and adaptive immune responses has not been conducted to date. In this study, we wished to advance on previous work and explored the association between dry eye manifestations and protein expression in tear fluid of pSS patients. Our findings suggest a correlation between severity of dry eye signs and upregulated levels of proteins in tear fluid from patients with pSS. Moreover, these overexpressed proteins showed an active involvement in both innate and adaptive immunity. Together, these findings can be useful in developing new non-invasive diagnostic methods, and assist in further monitoring disease progression.

## Materials and methods

### Study population

In the present study, 27 pSS patients fulfilling the American-European Consensus Group (AECG) classification criteria from 2002 [10] were recruited at the Department of Rheumatology, Oslo, University Hospital along with 32 age- and gender-matched controls recruited mainly at the Faculty of Dentistry, University of Oslo. The exclusion criteria for the healthy controls included a feeling of dryness in the mouth or eyes, presence of systemic disorders with oral or ocular involvement, and a history of surgical procedures that might affect secretion from the glands. Participants were referred to the Norwegian Dry Eye Clinic in Oslo. Upon enrolment, a detailed explanation of the protocols and study aim were given, and written informed consent was obtained from all participants. The study was approved by the Regional Medical Ethical Committee of South-East Norway (2015/363). Clinical data and medical records were acquired from patients’ records and through clinical examination at the Department of Rheumatology, Oslo University Hospital. This provided the data obtained through routine laboratory assessment, including evaluation of ocular dryness by assessment of tear secretion, and anti-Ro/SSA and anti-La/SSB. All patients included in the study had some residual secretory ability. The demographic data for the patients participating in the study is shown in **Table 1**, while detailed characteristics of the control group are given in **S1 Table**.

**Table 1:** Clinical characteristics of patients with pSS included in the study.

Study ID	Age (years)	Anti-SSA*	Anti-SSB*	OSDI**	Osmolarity*** (mOsm/L)	TFBUT**** (sec)	Schirmer’s ***** (mm)	Ocular surface staining ******	Corneal staining ******
**P1**	68	+	-	58.3	320	1	6.5	6	2.5
**P2**	40	+	+	56.3	327.5	1	14.5	0.5	0.5
**P3**	64	+	-	25	322.5	2.5	9.5	0	0
**P4**	32	+	-	29.2	363	3	13	5	1
**P5**	57	+	+	16.7	-	3	5	2	2
**P6**	55	+	+	40	344	4.5	5.5	1.5	1
**P7**	68	+	+	8.3	332.5	1.5	2.5	4	2.5
**P8**	39	+	+	54.2	-	15	-	3	1
**P9**	64	+	+	2.3	318.5	2.5	7.5	5.5	2.5
**P10**	72	+	+	16.7	352.5	1.5	-	2	2
**P11**	54	+	-	86.4	295.5	3	-	2.5	0
**P12**	36	+	-	10.4	-	1	0	8.5	3.5
**P13**	53	+	-	52.1	303	1.5	1	4.5	2
**P14**	47	+	+	32.5	366	1.5	5	3	1
**P15**	72	+	-	25	-	1	-	3.5	3.5
**P16**	54	+	+	35.4	314	7.5	13.5	0.5	0.5
**P17**	33	+	+	43.8	343	1	6	9	3
**P18**	68	+	+	43.8	307.5	1	0	4	3
**P19**	51	+	-	33.3	320.5	1	5	7.5	2.5
**P20**	48	+	+	39.6	353.5	1	1	5	4
**P21**	48	+	+	35.4	320.5	3.5	2	4	0.5
**P22**	44	+	+	30	321	5	4.5	3.5	0.5
**P23**	40	+	+	8.3	374.5	2	3.5	3.5	2
**P24**	57	+	+	45.8	346	1	1	4.5	2.5
**P25**	35	+	+	31.25	348.5	1	1.5	4	3
**P26**	71	+	-	31.25	319.5	1.5	7	4	2.5
**P27**	48	+	-	22.9	372.5	1.5	0	4	1.5

OSDI: Ocular Surface Disease Index; TFBUT: tear film break-up time

* Autoantibody production was assessed by ELISA

** Questionnaire (12 questions, score 0 to 100) to measure symptoms of ocular irritation related to DED; normal value ≤12

*** Diagnostic tool in DED with a normal value of <308 mOsm/L

**** Indicates tear film stability where values ≥10 sec are normal

***** Values are in mm/5 minutes; normal flow >10 mm/5 minutes

****** Used to evaluate ocular surface damage in potential DED. The Oxford grading scheme quantifies the estimated damage on a scale from 0 to 15. A higher score implies more ocular surface damage in exposed cornea and interpalpebral conjunctiva. Normal values for corneal staining and ocular surface staining are ≤1 and ≤3, respectively

### Clinical evaluation at the Norwegian Dry Eye Clinic

The dry eye examination was performed as previously described [25]. In brief, dry eye disease (DED) patients were required to answer the McMonnies Dry Eye (MDEIS) questionnaire and the Ocular Surface Disease Index (OSDI) questionnaire. Scores assessing subjective symptoms were thus attained. Thereafter, all participants underwent an extensive ophthalmic examination of both eyes, where average objective quantitative values were then registered. This included tear osmolarity measurement that uses the TearLab Osmolarity System (TearLab Corp, San Diego, CA) [26], which has been recognised as a clinical diagnostic tool in DED, where the threshold value ≥ 308 mOsm/L indicates dry eyes [27]. Tear film break-up time (TFBUT) measurement [28, 29] was also performed, followed by the assessment of tear production using the Schirmer’s test [28]. Moreover, ocular surface staining that was registered according to the Oxford grading scheme [30] was used to determine ocular surface damage in potential DED. Finally, corneal, temporal, and nasal bulbar conjunctival staining with fluorescein was also carried out and recorded according to the Oxford scoring scheme [30] (**Table 1** and **S1 Table**).

### Tear fluid collection and determination of protein amount

Tear fluid was collected using a Schirmer’s tear test strip (HAAG-STREIT, Essex, UK), followed by proteomics analysis performed on tear fluid from patients with pSS and controls, as described formerly [24]. In brief, in-solution protein digestion was carried out, followed by LC-MS using an Ultimate 3000 RSLCnano-UHPLC system connected to a Q Exactive mass spectrometer (Thermo Fisher Scientific, Bremen, Germany), further equipped with a nano electrospray ion source.

### Data processing

MS/MS-based peptide and protein identifications were validated using Scaffold (version Scaffold_4.4, Proteome Software Inc., Portland, OR, USA), as previously described [24]. Spectral counts for each protein were thus provided, and the five most upregulated protein in tear fluid of pSS patients could be identified based on our former proteomics data analysis [24]. Based on these former findings, the mean spectral counts were calculated for these five most upregulated proteins in tear fluid of pSS patients and controls in relation to the objective DED manifestations investigated (**S2 Table**). Moreover, patients were divided into two groups based on severity of eye examination parameters; namely non-pathological and pathological. The individuals within each of the non-pathological and pathological groups depend on the cut-off values for each eye examination parameter (**S1 Table**). The non-pathological pSS group represented measurements of dry eye examinations within the normal range, and could hence serve as an additional positive control. The DED manifestations accounted for include TFBUT (controls n = 11, non-pathological pSS n = 0, pathological pSS n = 11), Schirmer’s test (controls n = 10, non-pathological pSS n = 2, pathological pSS n = 9), ocular surface staining (controls n = 11, non-pathological pSS n = 2, pathological pSS n = 9), and corneal staining (controls n = 11, non-pathological pSS n = 6, pathological pSS n = 5). Furthermore, FunRich v3 (http://www.funrich.org/) was used to explore the biological processes of the 201 upregulated proteins that were previously detected via Scaffold in pSS patients compared to healthy controls using enrichment analysis.

### Statistical analysis

The Mann-Whitney U test was applied to determine whether there were any statistical significance between the different parameters among pSS patients and healthy controls.

## Results

### Increased severity of dry eye manifestations in pSS patients

**Fig 1:**
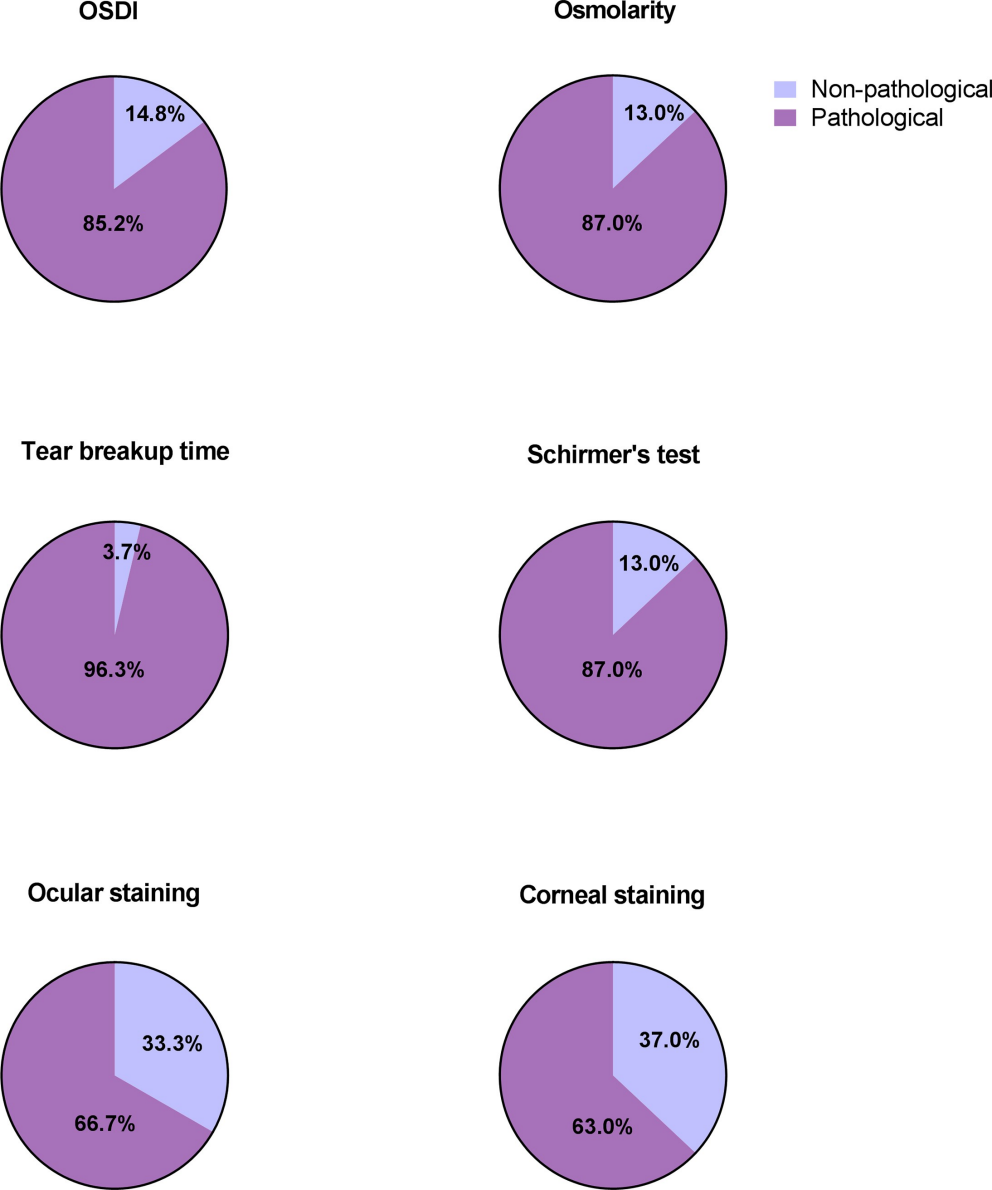
Increased severity of dry eye signs in pSS patients. Patients with pSS are divided into two groups based on severity of the eye examination parameters, namely non-pathological (light purple) and pathological (purple), where the non-pathological pSS group represents an additional positive control. This grouping demonstrates that the majority of pSS patients in our study exhibit pathological DED manifestations (OSDI 85.2%, osmolarity 87.0%, TFBUT 96.3%, Schirmer’s test 87.0%, ocular surface staining 66.7% and corneal staining 63.0%).

The OSDI questionnaire verified that most patients with pSS had DED symptoms, with the exception of P7, P9, P12, and P23. The mean values for OSDI, osmolarity, TFBUT, Schirmer’s test, ocular surface and corneal staining scores were 33.9±18.4, 334.2±22.5, 2.6±2.9, 5.0±4.3, 3.9±2.2, and 1.9±1.1, respectively, in the pSS patient group (**Table 1**). Meanwhile, the control group showed mean OSDI, osmolarity, TFBUT, Schirmer’s test, ocular surface and corneal staining scores of 4.8±7.5, 319.7±15.8, 5.4±3.3, 16.2±11.6, 0.8±1.2, and 0.3±0.5, correspondingly (**S1 Table**). Consequently, our eye examinations demonstrated significantly increased severity of tear osmolarity levels (p<0.0001), TFBUT (p<0.001), Schirmer’s test (p<0.001), ocular surface staining (p<0.001), and corneal staining (p<0.001) in pSS patients compared to healthy controls. Moreover, these pSS patients were divided into two groups, namely non-pathological and pathological, based on severity of the eye examination parameters. This division confirmed that the majority of pSS patients in our study exhibited pathological DED manifestations (**Fig 1**).

### Severity of dry eye signs correlates with levels of upregulated proteins in tear fluid from pSS patients

The proteome of tear fluid from patients with pSS and controls was previously examined by LC-MS [24]. The identification of proteins using the Mascot database search engine, and further data analysis using Scaffold were then applied. This helped identify quantitative differences based on t-test (p < 0.05), and provided the spectral count (i.e., abundance) for each protein in the pSS group compared to controls. The five most upregulated proteins detected in pSS patients included DNA (apurinic or apyrimidinic site) lyase (APEX1), thioredoxin-dependent peroxidase reductase (PRDX3), copine (CPNE1), aconitate hydratase (ACO2), and LIM domain only protein 7 (LMO7), in descending order [24].

**Fig 2:**
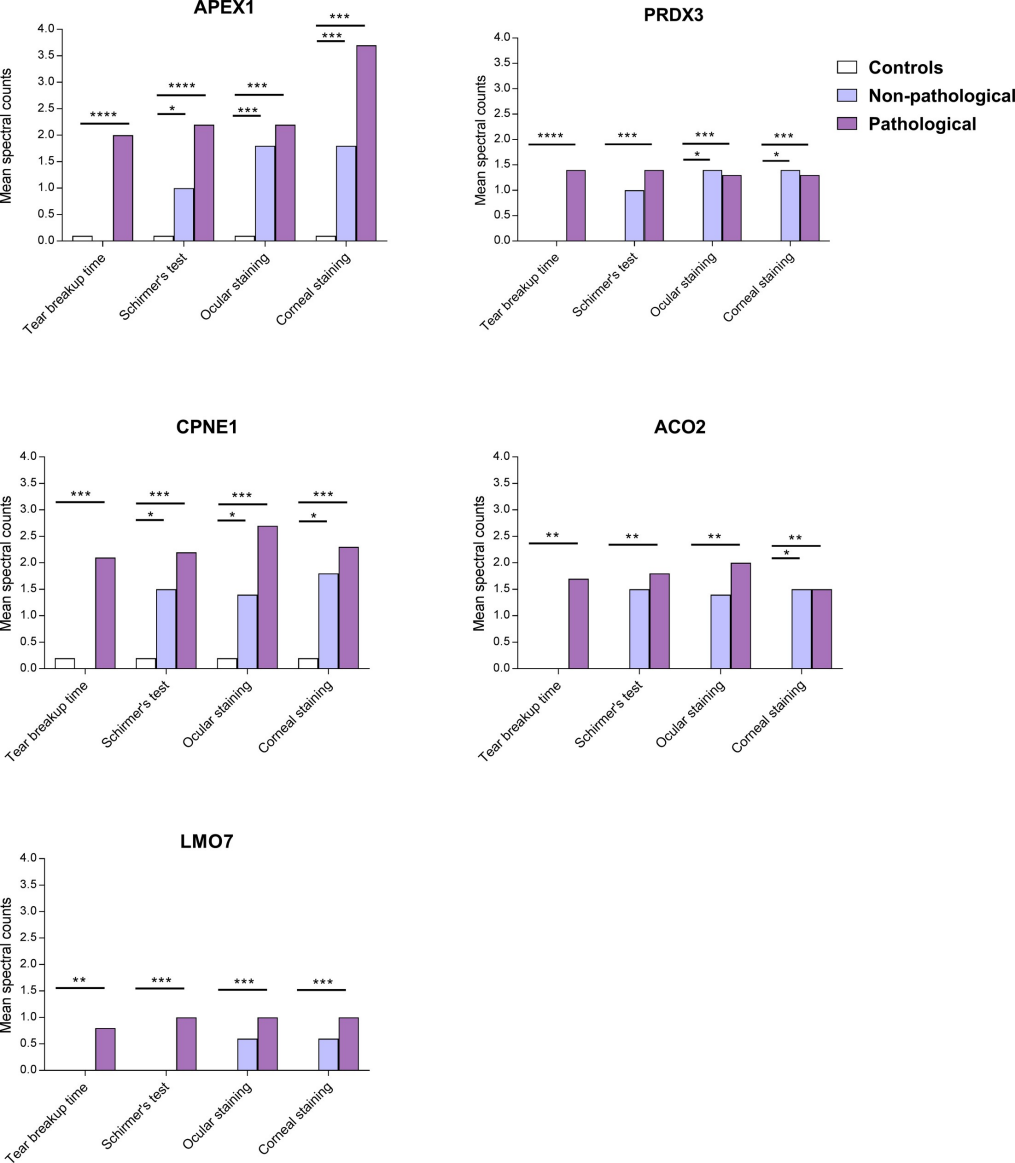
Severity of dry eye signs correlates with levels of upregulated proteins in tear fluid from pSS patients. Mean spectral counts are calculated for upregulated proteins in tear fluid, in controls (white) non-pathological pSS patients (light purple), and pathological pSS patients (purple), exploring the DED signs TFBUT, Schirmer’s test, ocular surface staining, and corneal staining. **A)** Patients with pSS expressing pathological DED manifestations show greatest levels of APEX1, compared to both the non-pathological pSS patient group and the controls. There remain higher levels of protein in the non-pathological pSS group when compared to the healthy controls for all DED manifestations except TFBUT. **B)** PRDX3 is not observed in the control group, while highest levels of protein are detected in the pathological pSS group for TFBUT and Schirmer’s test. Greater abundance of PRDX3 is observed in the non-pathological pSS group with regard to ocular staining and corneal staining. **C)** Greatest levels of CPNE1 are seen in the pathological severity group of pSS for all DED manifestations. Higher levels of protein are found in the non-pathological pSS group when compared to healthy controls, with the exception of TFBUT. **D)** ACO2 is mostly expressed in the pathological group of pSS patients for the DED signs explored, except for corneal staining, where a similar amount of protein is also observed in the non-pathological patient group. No protein is detected in the control group. **E)** LMO7 is most abundant in the pathological patient group for all DED signs. No protein is observed in the control group for all signs, and for TFBUT and Schirmer’s test in the non-pathological pSS group. Significant p-values are indicated by: * p < 0.05, ** p < 0.01, *** p < 0.001, **** p < 0.0001.

The mean spectral counts were then calculated for these five most upregulated proteins in tear fluid based on our former proteomics data analysis [24]. Here, patients were divided into two groups based on severity of eye examination parameters (non-pathological and pathological), as described above. Our results show a general increase in mean spectral counts for all five proteins in the pSS patients compared to the healthy controls, where an even greater abundance of protein was observed in the group of pSS patients exhibiting pathological DED manifestations (**S2 Table**). A visualisation of the distribution of mean spectral counts (i.e., protein abundance) for the aforementioned DED signs, with regard to each of the five upregulated proteins in our study groups, was then generated. This provided an overview of the effect of DED manifestations on each protein level in controls, non-pathological and pathological pSS patients (**Fig 2**). Patients with pSS expressing pathological DED manifestations showed significantly higher levels of APEX1, compared to the healthy controls (p < 0.0001). Meanwhile, there remains a higher level of protein in the non-pathological pSS group when compared to the healthy controls for all DED signs, except TFBUT (p < 0.001) (**Fig 2A**). Interestingly, PRDX3 was not detected in the control group. However, significantly increased levels of PRDX3 were found in the pathological pSS group for TFBUT and Schirmer’s test (p < 0.001), while greater abundance of PRDX3 was observed in the non-pathological pSS group with regard to ocular staining and corneal staining (p < 0.05) (**Fig 2B**). Similar to APEX1, the trend seen for CPNE1 demonstrated greatest levels of protein in the pathological group of pSS, for all DED signs when compared to healthy controls (p < 0.001). Again, higher levels of protein were detected in the non-pathological pSS group when compared to healthy controls (p < 0.05), with the exception of TFBUT (**Fig 2C**). Furthermore, ACO2 was also mostly expressed in the pathological group of pSS patients for the DED manifestations explored (p < 0.01), except for corneal staining, where a similar amount of protein was also found in the non-pathological patient group. No protein was detected in the control group (**Fig 2D**). Lastly, LMO7 was most abundant in the pathological pSS patient group for all DED signs (p < 0.001). Surprisingly, no protein was identified in the control group for all signs, and for TFBUT and Schirmer’s test in the non-pathological patient group (**Fig 2E**).

### Overexpression of proteins involved in innate and adaptive immune responses detected in tear fluid from pSS patients

**Fig 3:**
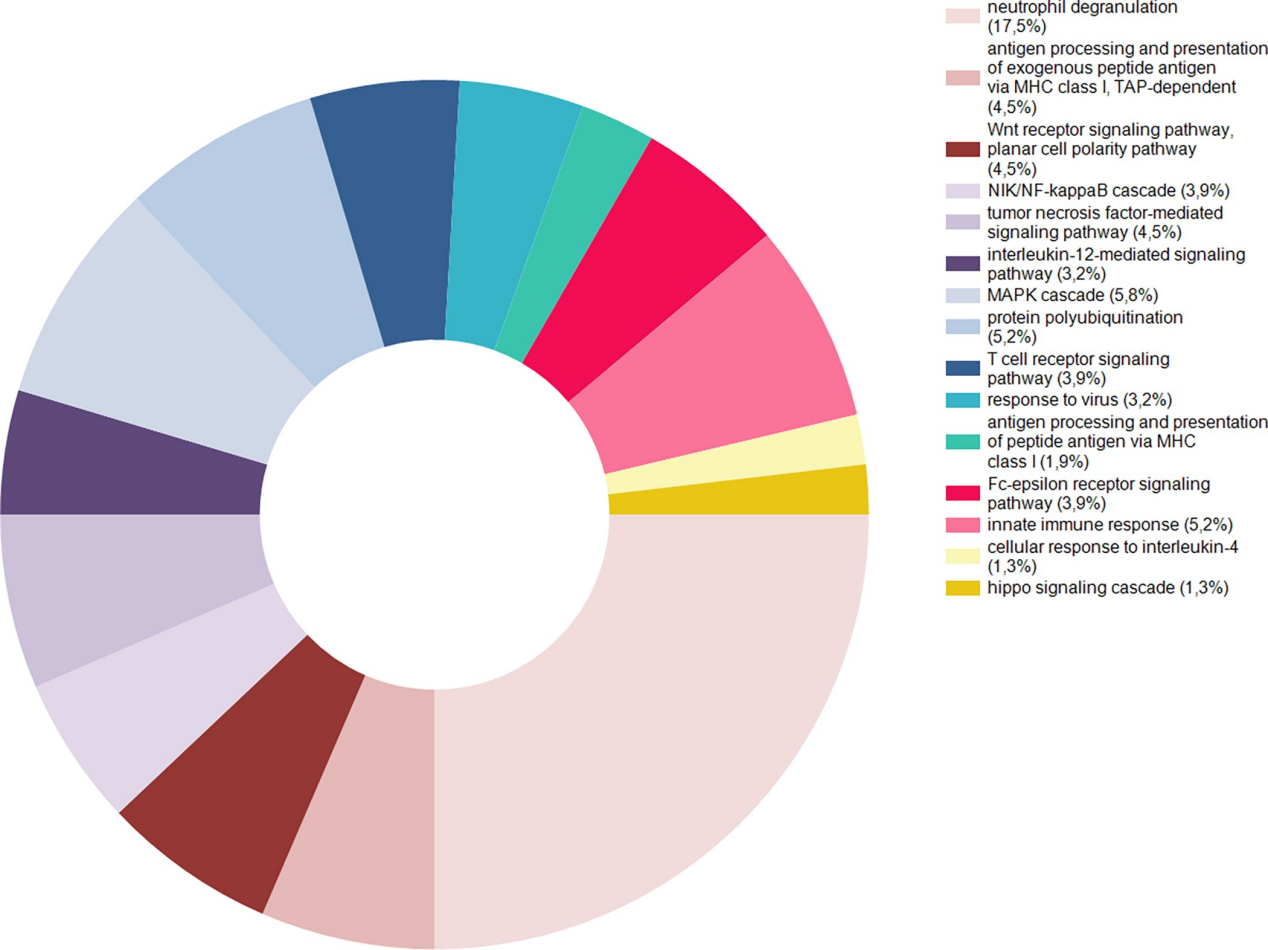
Overexpression of proteins involved in innate and adaptive immune responses detected in tear fluid from pSS patients. The cellular pathways identified in tear fluid of pSS patients are active components of either innate and/or adaptive immunity, and involve cellular processes that include neutrophil degranulation, antigen processing presentation (MHC class I), Wnt receptor signalling pathway, NF-kappaB cascade, TNF mediated signalling, IL-12 mediated signalling, MAP kinase cascade, protein polyubiquitination, T cell receptor signalling, innate immune responses, cellular response to IL-4, and the hippo signalling cascade. FunRich v3 (http://www.funrich.org/) was used to explore the biological processes of the upregulated proteins.

FunRich v3 (http://www.funrich.org/) was used to explore the biological processes of the 201 upregulated proteins detected formerly in pSS patients compared to healthy controls. The cellular pathways identified were active components of either innate and/or adaptive immunity. These included neutrophil degranulation, antigen processing presentation (MHC class I), Wnt receptor signalling pathway, NF-κB cascade, TNF mediated signalling, IL-12 mediated signalling, MAP kinase cascade, protein polyubiquitination, T cell receptor signalling, innate immune responses, cellular response to IL-4, and the hippo signalling cascade (**Fig 3**).

## Discussion

Regulated tear production is necessary for maintaining a healthy ocular surface. Therefore, a disturbance in this regulated process due to hyposecretion of tear components may cause increased local evaporation of the tear fluid. This could consequently result in dryness, making the ocular surface epithelium more prone to damage. Such disturbances in tear production, as evaluated by Schirmer’s test and TFBUT, have been detected in the majority of pSS patients included in our present analysis (**Fig 1**) [31]. Furthermore, implications of less stable tear film, due to decreased lacrimal secretion, may lead to damage and subsequent destruction of conjunctival and corneal epithelial cells, in particular. Routine ocular surface staining can therefore aid in evaluating severity of ocular surface inflammation in these patients, and in turn provide surface staining scores, as accounted for in our patient group (**Fig 1**) [32–34]. Interestingly, the prevalence of DED in the general population is relatively high [35]. This occurrence can be observed in our healthy control group also demonstrating dry eye manifestations (**S1 Table**). Taking all this into account, such disturbances in lacrimal secretion are consequent characteristic features of pSS [36]. Clearly, these routine assessments of ocular dryness and ocular surface inflammation explored in this study could be viewed as helpful tools in not only classifying, but also monitoring the disease. Such measurements could also be considered as potential indicators of disease progression in pSS.

An additional non-invasive method for diagnosing and monitoring pSS could involve studying the proteome of biological fluids through LC-MS approaches. By applying such techniques, the search for potential biomarkers can be realised for pSS. Saliva has thus far been the biological fluid used in the majority of previous proteomic studies, where different mass spectrometry approaches and genomics were applied [15–21]. Meanwhile, tear fluid has only been used in a limited number of proteomic studies when identifying potential biomarkers for pSS [22–24]. Hence, our previous study focused on applying LC-MS on tear fluid collected from patients with pSS, allowing potential biomarkers in lacrimal disease target organs to be identified [24].

Using Scaffold, the five most upregulated proteins in pSS patients were identified in tear fluid. These proteins are involved in a broad range of cellular functions, involving oxidative stress (APEX1), B cell survival (PRDX3), TNF-α signalling (CPNE1), the Krebs cycle (ACO2), and cell adhesion and ubiquitination (LMO7).

To advance on previous findings, the mean spectral counts were calculated for the aforementioned upregulated proteins in tear fluid in relation to DED manifestations [24]. Generally, our current results demonstrated an increase in mean spectral counts for all five proteins in pSS patients compared to healthy controls, where an even greater abundance of protein was observed in the pathological group of pSS patients for all DED manifestations explored (**S2 Table**). Visualising the distribution of mean spectral counts for these DED signs, with regard to each of the five upregulated proteins in our study groups, provided an overview of the effect of DED manifestations on each protein level (**Fig 2**). Here, patients with pSS expressing pathological DED manifestations showed significantly greater levels of proteins compared to both the non-pathological pSS patient group and the healthy controls in most instances. Interestingly, greatest abundance of PRDX3 was observed in the non-pathological pSS group with regard to ocular staining and corneal staining (**Fig 2B**). Meanwhile, PRDX3, ACO2, and LMO7 were not detected in the control groups for all signs, suggesting that these proteins could be expressed as a result of disease activity (**Fig 2B, 2D and 2E**). Overall, these observations demonstrate the effect of DED on levels of upregulated proteins in pSS, where the presence of DED manifestations results in a subsequent increase in protein expression.

In order to delineate cellular pathways involving the upregulated proteins identified in tear fluid with LC-MS, DAVID analysis and FunRich v3 were applied. The biological processes affected included neutrophil degranulation, TNF mediated signalling, antigen processing presentation (MHC class I), Wnt receptor signalling pathway, NF-kappaB cascade, IL-12 mediated signalling, MAP kinase cascade, protein polyubiquitination, T cell receptor signalling, cellular response to IL-4, and the hippo signalling cascade (**Fig 3**). Viewed as a whole, these identified cellular pathways and components clearly indicate the involvement of autoimmune reactions and over-activation of the innate and adaptive immune systems in patients with pSS, both as a consequence of disease pathogenesis and probably also as part of the healing process.

## Conclusions

In summary, by conducting a thorough analysis of the relationship between clinical dry eye signs and recently suggested biomarkers in pSS, we could explore how this in turn relates to disturbances in innate and adaptive immune responses. Our observations suggest a possible relationship between presence of dry eye manifestations and upregulated proteins in tear fluid from patients with pSS. Moreover, these overexpressed proteins show an active involvement in both innate and adaptive immunity. Together, these findings highlight the importance of routine assessments of ocular dryness and ocular surface inflammation explored in this study. Further studies are needed to verify the concepts explored and analyses performed in a greater cohort of pSS patients, where sensitivity and specificity of the methods conducted could also be substantiated.

## Supporting information

S1 Table Clinical characteristics of healthy controls included in the study. OSDI: Ocular Surface Disease Index; TFBUT: tear film break-up time.
* Autoantibody production was assessed by ELISA
** Questionnaire (12 questions, score 0 to 100) to measure symptoms of ocular irritation related to DED; normal value ≤12
*** Diagnostic tool in DED with a normal value of <308 mOsm/L
**** Indicates tear film stability where values ≥10 sec are normal
***** Values are in mm/5 minutes; normal flow >10 mm/5 minutes
****** Used to evaluate ocular surface damage in potential DED. The Oxford grading scheme quantifies the estimated damage on a scale from 0 to 15. A higher score implies more ocular surface damage in exposed cornea and interpalpebral conjunctiva. Normal values for corneal staining and ocular surface staining are ≤1 and ≤3, respectively.
(DOCX)

S2 Table Mean spectral counts of upregulated proteins in tear fluid in relation to clinical eye manifestations. The mean spectral counts were calculated for the five most upregulated proteins in tear fluid of pSS patients and controls in relation to the objective DED manifestations investigated. Patients were divided into two groups based on severity of eye examination parameters; namely non-pathological and pathological. The individuals within each of the non-pathological and pathological groups depend on the cut-off values for each eye examination parameter. The non-pathological pSS group represented measurements of dry eye examinations within the normal range, and could hence serve as an additional positive control. The DED manifestations accounted for include TFBUT (controls n = 11, non-pathological pSS n = 0, pathological pSS n = 11), Schirmer’s test (controls n = 10, non-pathological pSS n = 2, pathological pSS n = 9), ocular surface staining (controls n = 11, non-pathological pSS n = 2, pathological pSS n = 9), and corneal staining (controls n = 11, non-pathological pSS n = 6, pathological pSS n = 5).
(DOCX)

S1 File Identification of potential saliva and tear biomarkers in primary Sjogren's syndrome, utilising the extraction of extracellular vesicles and proteomics analysis. Aqrawi LA, Galtung HK, Vestad B, Ovstebo R, Thiede B, Rusthen S, et al. Arthritis Res Ther. 2017 Jan 25;19(1):14. PubMed PMID: 28122643. Pubmed Central PMCID: 5264463.
(PDF)

S2 File Interdisciplinary, comprehensive oral and ocular evaluation of Patients with primary Sjogren's syndrome. Tashbayev B, Rusthen S, Young A, Herlofson BB, Hove LH, Singh PB, et al. Scientific reports. 2017 Sep 7;7(1):10761. PubMed PMID: 28883442. Pubmed Central PMCID: 5589846.
(PDF)
