# Supplementary material for: Severity of clinical dry eye manifestations influences protein expression in tear fluid of patients with primary Sjögren’s syndrome
Source: PLoS One. 2018 Oct 12;13(10):e0205762. doi: 10.1371/journal.pone.0205762 (PMC6185846; doi:10.1371/journal.pone.0205762)
Supplement: S1 Table — OSDI: Ocular Surface Disease Index; TFBUT: tear film break-up time. * Autoantibody production was assessed by ELISA ** Questionnaire (12 questions, score 0 to 100) to measure symptoms of ocular irritation related to DED; normal value ≤12 *** Diagnostic tool in DED with a normal value of <308 mOsm/L **** Indicates tear film stability where values ≥10 sec are normal ***** Values are in mm/5 minutes; normal flow >10 mm/5 minutes ****** Used to evaluate ocular surface damage in potential DED. The Oxford grading scheme quantifies the estimated damage on a scale from 0 to 15. A higher score implies more ocular surface damage in exposed cornea and interpalpebral conjunctiva. Normal values for corneal staining and ocular surface staining are ≤1 and ≤3, respectively. (DOCX) [file pone.0205762.s001.docx]

**S1 Table.** Clinical characteristics of healthy controls included in the study

| Study ID | Age  (years) | Anti-SSA* | Anti-SSB* | OSDI** | Osmolarity*** (mOsm/L) | TBUT****  (sec) | Schirmer´s *****  (mm) | Ocular surface staining ****** | Corneal staining ****** |
| --- | --- | --- | --- | --- | --- | --- | --- | --- | --- |
| C1 | 50 | - | - | 2.00 | 309.50 | 3.50 | 27.50 | 0.00 | 0.00 |
| C2 | 57 | - | - | 0.00 | 316.00 | 5.00 | 16.00 | 0.50 | 0.50 |
| C3 | 54 | - | - | 0.00 | 313.50 | 2.00 | 10.50 | 1.50 | 0.50 |
| C4 | 52 | - | - | 2.00 | 337.00 | 7.50 | 20.50 | 2.00 | 1.00 |
| C5 | 40 | - | - | 2.08 | 300.50 | 6.50 | 32.00 | 0.00 | 0.00 |
| C6 | 63 | - | - | 4.16 | 313.50 | 6.00 | 17.50 | 0.00 | 0.00 |
| C7 | 34 | - | - | 2.08 | 323.00 | 4.00 | 35.00 | 0.50 | 0.00 |
| C8 | 44 | - | - | 2.08 | 321.00 | 9.00 | 6.00 | 0.00 | 0.00 |
| C9 | 34 | - | - | 0.00 | 336.00 | 15.00 | 26.00 | 0.00 | 0.00 |
| C10 | 54 | - | - | 0.00 | 347.50 | 9.00 | 4.50 | 0.00 | 0.00 |
| C11 | 35 | - | - | 0.00 | 317.50 | 9.00 | 32.50 | 3.00 | 1.00 |
| C12 | 50 | - | - | 2.08 | 320.00 | 6.00 | - | 0.00 | 0.00 |
| C13 | 57 | - | - | 2.08 | 306.00 | 3.00 | 16.50 | 0.00 | 0.00 |
| C14 | 57 | - | - | 8.33 | 325.50 | 3.00 | 8.00 | 0.00 | 0.00 |
| C15 | 40 | - | - | 5.00 | 317.50 | 3.50 | 35.00 | 0.00 | 0.00 |
| C16 | 51 | - | - | 8.33 | 300.00 | 3.00 | 1.00 | 1.00 | 1.00 |
| C17 | 73 | - | - | 6.25 | 334.50 | 2.00 | 10.00 | 0.00 | 0.00 |
| C18 | 33 | - | - | 0.00 | 303.00 | 7.00 | - | 0.00 | 0.00 |
| C19 | 46 | - | - | 39.58 | 307.50 | 13.00 | 35.00 | 0.00 | 0.00 |
| C20 | 41 | - | - | 4.17 | 295.50 | 2.00 | 0.00 | 0.00 | 0.00 |
| C21 | 44 | - | - | 4.17 | 340.00 | 8.50 | 14.50 | 0.00 | 0.00 |
| C22 | 32 | - | - | 8.33 | 317.00 | 8.50 | 4.50 | 2.50 | 0.00 |
| C23 | 43 | - | - | 2.70 | - | 7.00 | - | 5.50 | 0.00 |
| C24 | 50 | - | - | 18.75 | 312.50 | 3.00 | 5.00 | 1.50 | 0.00 |
| C25 | 43 | - | - | 6.25 | 299.50 | 4.00 | 5.00 | 0.50 | 0.00 |
| C26 | 47 | - | - | 2.08 | 307.00 | 7.00 | 2.50 | 0.50 | 0.50 |
| C27 | 46 | - | - | 0.00 | 304.00 | 1.00 | 30.00 | 2.00 | 0.00 |
| C28 | 54 | - | - | 10.42 | 358.00 | 3.00 | 25.50 | 0.00 | 0.00 |
| C29 | 79 | - | - | 4.17 | 341.00 | 1.00 | 8.00 | 1.00 | 1.00 |
| C30 | 69 | - | - | 4.17 | 336.50 | 6.00 | 18.00 | 1.50 | 0.50 |
| C31 | 38 | - | - | 2.08 | 334.50 | 2.50 | 3.00 | 2.00 | 2.00 |
| C32 | 58 | - | - | 0.00 | 316.00 | 3.50 | 19.00 | 0.00 | 0.00 |

OSDI: Ocular Surface Disease Index; TFBUT: tear film break-up time

* Autoantibody production was assessed by ELISA
** Questionnaire (12 questions, score 0 to 100) to measure symptoms of ocular irritation related to DED; normal value ≤12

*** Diagnostic tool in DED with a normal value of <308 mOsm/L

**** Indicates tear film stability where values ≥10 sec are normal

***** Values are in mm/5 minutes; normal flow >10 mm/5 minutes

****** Used to evaluate ocular surface damage in potential DED. The Oxford grading scheme quantifies the estimated damage on a scale from 0 to 15. A higher score implies more ocular surface damage in exposed cornea and interpalpebral conjunctiva. Normal values for corneal staining and ocular surface staining are ≤1 and ≤3, respectively
