# Supplementary material for: Severity of clinical dry eye manifestations influences protein expression in tear fluid of patients with primary Sjögren’s syndrome
Source: PLoS One. 2018 Oct 12;13(10):e0205762. doi: 10.1371/journal.pone.0205762 (PMC6185846; doi:10.1371/journal.pone.0205762)
Supplement: S2 Table — The mean spectral counts were calculated for the five most upregulated proteins in tear fluid of pSS patients and controls in relation to the objective DED manifestations investigated. Patients were divided into two groups based on severity of eye examination parameters; namely non-pathological and pathological. The individuals within each of the non-pathological and pathological groups depend on the cut-off values for each eye examination parameter. The non-pathological pSS group represented measurements of dry eye examinations within the normal range, and could hence serve as an additional positive control. The DED manifestations accounted for include TFBUT (controls n = 11, non-pathological pSS n = 0, pathological pSS n = 11), Schirmer’s test (controls n = 10, non-pathological pSS n = 2, pathological pSS n = 9), ocular surface staining (controls n = 11, non-pathological pSS n = 2, pathological pSS n = 9), and corneal staining (controls n = 11, non-pathological pSS n = 6, pathological pSS n = 5). (DOCX) [file pone.0205762.s002.docx]

**S2 Table.** Mean spectral counts of upregulated proteins in tear fluid in relation to clinical eye manifestations.

*A. Tear breakup time*

| **Upregulated protein** | **Controls** | **Non-pathological pSS** | **Pathological pSS** |
| --- | --- | --- | --- |
| APEX1 | 0.1 | - | 2 |
| PRDX3 | 0 | - | 1.4 |
| CPNE1 | 0.2 | - | 2.1 |
| ACO2 | 0 | - | 1.7 |
| LMO7 | 0 | - | 0.8 |

*B. Schirmer’s test*

| **Upregulated protein** | **Controls** | **Non-pathological pSS** | **Pathological pSS** |
| --- | --- | --- | --- |
| APEX1 | 0.1 | 1 | 2.2 |
| PRDX3 | 0 | 1 | 1.4 |
| CPNE1 | 0.2 | 1.5 | 2.2 |
| ACO2 | 0 | 1.5 | 1.8 |
| LMO7 | 0 | 0 | 1 |

*C. Ocular staining*

| **Upregulated protein** | **Controls** | **Non-pathological pSS** | **Pathological pSS** |
| --- | --- | --- | --- |
| APEX1 | 0.1 | 1.8 | 2.2 |
| PRDX3 | 0 | 1.4 | 1.3 |
| CPNE1 | 0.2 | 1.4 | 2.7 |
| ACO2 | 0 | 1.4 | 2 |
| LMO7 | 0 | 0.6 | 1 |

*D. Corneal staining*

| **Upregulated protein** | **Controls** | **Non-pathological pSS** | **Pathological pSS** |
| --- | --- | --- | --- |
| APEX1 | 0.1 | 1.8 | 3.7 |
| PRDX3 | 0 | 1.4 | 1.3 |
| CPNE1 | 0.2 | 1.8 | 2.3 |
| ACO2 | 0 | 1.5 | 1.5 |
| LMO7 | 0 | 0.6 | 1 |
