# Supplementary material for: Severity of clinical dry eye manifestations influences protein expression in tear fluid of patients with primary Sjögren’s syndrome
Source: PLoS One. 2018 Oct 12;13(10):e0205762. doi: 10.1371/journal.pone.0205762 (PMC6185846; doi:10.1371/journal.pone.0205762)
Supplement: S1 File — Aqrawi LA, Galtung HK, Vestad B, Ovstebo R, Thiede B, Rusthen S, et al. Arthritis Res Ther. 2017 Jan 25;19(1):14. PubMed PMID: 28122643. Pubmed Central PMCID: 5264463. (PDF) [file pone.0205762.s003.pdf]

Aqrawi et al. Arthritis Research & Therapy (2017) 19:14
DOI 10.1186/s13075-017-1228-x

Arthritis Research & Therapy

RESEARCH ARTICLE Open Access

Identification of potential saliva and tear biomarkers in primary Sjögren's syndrome, utilising the extraction of extracellular vesicles and proteomics analysis

Lara A. Aqrawi[1*], Hilde Kanli Galtung[2], Beate Vestad[3], Reidun Øvstebø[3], Bernd Thiede[4], Shermin Rusthen[1], Alix Young[5], Eduarda M. Guerreiro[2], Tor Paaske Utheim[2,3,6], Xiangjun Chen[6], Øygunn Aass Utheim[3,7], Øyvind Palm[8] and Janicke Liaaen Jensen[1]

Abstract

Background: There is a long-lasting need for non-invasive, more accurate diagnostic techniques when evaluating primary Sjögren's syndrome (pSS) patients. Incorporation of additional diagnostics involving screening for disease-specific biomarkers in biological fluid is a promising concept that requires further investigation. In the current study we aimed to explore novel disease biomarkers in saliva and tears from pSS patients.

Methods: Liquid chromatography-mass spectrometry (LC-MS) was performed on stimulated whole saliva and tears from 27 pSS patients and 32 healthy controls, and salivary and tear proteomic biomarker profiles were generated. LC-MS was also combined with size exclusion chromatography to isolate extracellular vesicles (EVs) from both fluids. Nanoparticle tracking analysis was conducted on joint fractions from the saliva and tears to determine size distribution and concentration of EVs. Further EV characterisation was performed by immunoaffinity capture of CD9-positive EVs using magnetic beads, detected by flow cytometry. The LC-MS data were analysed for quantitative differences between patient and control groups using Scaffold, and the proteins were further analysed using the Database for Annotation, Visualization and Integrated Discovery (DAVID), for gene ontology overrepresentation, and the Search Tool for the Retrieval of Interacting Genes/Proteins for protein-protein interaction network analysis.

Results: Upregulation of proteins involved in innate immunity (LCN2), cell signalling (CALM) and wound repair (GRN and CALML5) were detected in saliva in pSS. Saliva EVs also displayed biomarkers critical for activation of the innate immune system (SIRPA and LSP1) and adipocyte differentiation (APMAP). Tear analysis indicated overexpression of proteins involved in TNF-α signalling (CPNE1) and B cell survival (PRDX3). Moreover, neutrophil gelatinase-associated lipocalin was upregulated in saliva and tears in pSS. Consistently, DAVID analysis demonstrated pathways of the adaptive immune response in saliva, of cellular component assembly for saliva EVs, and of metabolism and protein folding in tears in pSS patients.

Conclusions: LC-MS of saliva and tears from pSS patients, solely and in combination with size-exclusion chromatography allowed screening for possible novel biomarkers encompassing both salivary and lacrimal disease target organs. This approach could provide additional diagnostic accuracy in pSS, and could possibly also be applied for staging and monitoring the disease.

Keywords: Sjögren's syndrome, Autoimmunity, Inflammation, Innate immunity, Adaptive immunity, Saliva, Tears, Proteomics, Extracellular vesicles, Biomarkers

* Correspondence: l.a.aqrawi@odont.uio.no
[1]Department of Oral Surgery and Oral Medicine, Faculty of Dentistry, University of Oslo, Oslo, Norway
Full list of author information is available at the end of the article

© The Author(s). 2017 **Open Access** This article is distributed under the terms of the Creative Commons Attribution 4.0 International License (http://creativecommons.org/licenses/by/4.0/), which permits unrestricted use, distribution, and reproduction in any medium, provided you give appropriate credit to the original author(s) and the source, provide a link to the Creative Commons license, and indicate if changes were made. The Creative Commons Public Domain Dedication waiver (http://creativecommons.org/publicdomain/zero/1.0/) applies to the data made available in this article, unless otherwise stated.

Background

Sjögren's syndrome (SS) is a systemic rheumatic autoimmune disease, where chronic inflammation results in progressive destruction of exocrine glands, primarily the lacrimal and salivary glands [1, 2]. Thus, characteristic features are sicca symptoms, including dry eyes and dry mouth [3]. The prevalence of SS has been reported to be between 0.01% and 0.6% [4–6].

The main classification criteria used today when diagnosing primary SS (pSS) are the American-European Consensus Group (AECG) criteria from 2002 [7], which rely on evaluating symptoms of ocular and oral dryness, assessing the secretory ability of the exocrine glands, screening for anti-Ro and anti-La autoantibodies, and evaluating biopsies of minor salivary glands for mononuclear cell infiltration [8]. This routine assessment of minor salivary gland tissue and histological focus scoring has been employed to describe salivary gland involvement in SS [9, 10]. Here, a positive biopsy with mononuclear cell infiltrates comprising ≥50 mononuclear cells per 4 mm^2 resulted in a positive focus score value ranging from 1 to 12 according to the number of foci seen. This is a semi-quantitative, invasive technique useful for patients with glandular dysfunctions without autoantibody production [11].

Considering the nature of the currently available diagnostic tools, there remains an unmet need for non-invasive, more accurate diagnosis of pSS. The incorporation of additional non-invasive diagnostics, such as screening for disease-specific biomarkers [12, 13] has therefore been in focus over recent decades, as it can also be applied for staging and monitoring of the disease. Indeed, liquid chromatography-mass spectrometry (LC-MS) has been applied in several human rheumatic diseases, including SS, in order to discover biomarkers and therapeutic targets by studying the proteome of biological fluids [14, 15]. Both saliva [14, 16–21] and tear fluid [22, 23] have previously been used to identify potential biomarkers for SS. It has been reported that oral fluid not only reflects the salivary gland involvement that characterises SS disease [18, 24, 25], but also has the potential to represent the subject's current general health [26, 27]. Moreover, salivary fluid samples can easily be obtained using a non-invasive, simple, safe, and stress-free procedure, allowing for repetition and multiple collections. This explains why the majority of proteomic studies of SS have chosen saliva as the ideal biological fluid, examining either whole saliva or saliva from individual glands (e.g. minor and/or parotid salivary glands), under both stimulated and unstimulated conditions [14, 16–21]. As a result, several common biomarkers for SS have been found, including secretory proteins, enzymes, highly abundant immune-system-related molecules (e.g. β2-microglobulin), and cytokines such as IL-4 and IL-5 [21, 28, 29].

Proteomic analyses can also be coupled with various separation techniques in order to isolate the cellular components of interest when screening for disease biomarkers. Extracellular vesicles (EVs) are an example of such cellular components. These are membrane-embedded vesicles, comprising exosomes (size <100 nm) and/or microvesicles (size 100–1000 nm) [30], released by cells that are emerging as important mediators of intercellular communication, and thereby influencing recipient cell functions [31–33]. For instance, EVs can act on the innate immune system as paracrine messengers and have been described as pro-inflammatory mediators that induce inflammatory signals during infections [34, 35] and chronic inflammatory diseases [35].

Interestingly, patients with autoimmune diseases have increased levels of EVs that carry components associated with complement activation [36, 37]. Accordingly, various cell types of the innate immune system are known to release EVs, including macrophages [38], monocytes or dendritic cells [39] and natural killer (NK) cells [40]. Besides mediating the exchange of intercellular information by their surface molecules, EVs have been shown to be carriers of important soluble mediators, such as cytokines. The involvement of EVs in the transport of the cytokines IL-1b [41] and tumour necrosis factor (TNF) [42] are such examples.

Proteomic profiling of EVs in a biological context can be challenging, especially if the EV preparations are not highly purified [43]. In complex body fluids, EVs can be separated from interfering molecules, such as proteins and lipids, by utilising size-exclusion chromatography [44, 45]. The isolated sub-fractions containing the highest EV concentrations can then be characterised using nanoparticle tracking analysis, and by flow cytometry detection of the fusogenic protein/tetraspanin CD9, which is abundantly expressed in EVs [46–48].

Proteomic studies of isolated EVs have in turn yielded extensive catalogues that display which proteins are abundant in different types of EVs, specifically reflecting vesicle localization, cellular origin, and mechanism of secretion [49]. Hence, in the current study we hypothesised that by applying LC-MS alone, and in combination with EV-isolation, using samples of stimulated whole saliva and tear fluid from patients with pSS and healthy controls, novel biomarkers may be identified encompassing both salivary and lacrimal disease target organs. Such biomarkers may in turn be implemented, as potential non-invasive diagnostic tools that can help to increase diagnostic accuracy when evaluating patients with pSS, in accordance with the AECG criteria, and can also be useful when monitoring disease progression.

Methods

Study population

Patients with pSS (n = 27) that fulfilled the AECG classification criteria from 2002 [7] and 32 age-matched and gender-matched controls participated in this study. Following recruitment at the Department of Rheumatology, Oslo

University Hospital, the patients were referred to the Dry Mouth Clinic, located at the Institute of Clinical Dentistry, Faculty of Dentistry, University of Oslo, and the Norwegian Dry Eye Clinic, Oslo, for thorough examination and sample collection, as described below. A detailed explanation of the study aim and protocols were explained to the recruited subjects upon enrolment. Written informed consent was obtained from the participants and the Regional Medical Ethical Committee of South-East Norway approved the study (2015/363).

Medical records and clinical data were obtained through clinical examination and from patients' charts at the Department of Rheumatology, Oslo University Hospital. This provided information that had been collected during routine laboratory assessments, including anti-Ro/SSA and anti-La/SSB, and evaluation of ocular and oral dryness by assessing saliva and tear secretion. Some residual secretory ability was required for inclusion of the patients in the study. The demographic data for the patients included in this study are presented in Table 1.

Table 1 Clinical characteristics of patients with pSS included in the study

ID number	Age (years)	Anti-SSA[b]	Anti-SSB[b]	Schirmer test[c]	Saliva secretion[d]	Dry mouth	Dry eyes
PSS1	69	+	-	+	+	+	+
PSS2	41	+	+	-	+	+	+
pSS3	64	+	-	-	+	+	+
pSS4	33	+	-	+	+	+	-
pSS5	57	+	+	+	+	+	+
pSS6	55	+	+	+	+	+	+
pSS7	69	+	+	+	+	+	+
pSS8	40	+	+	+	+	+	+
pSS9	64	+	+	+	+	+	+
pSS10	72	+	+	+	+	+	+
pSS11	54	+	-	+	+	+	+
pSS12	36	+	-	+	+	+	+
pSS13	53	+	-	+	+	+	+
pSS14	47	+	+	+	+	+	+
pSS15	73	+	-	+	-	+	+
pSS16	54	+	+	+	+	+	+
pSS17	33	+	+	+	+	+	+
pSS18	69	+	+	+	+	+	+
pSS19	51	+	-	+	-	+	+
pSS20	48	+	+	+	+	+	+
pSS21[a]	48	+	+	+	+	+	+
pSS22[a]	44	+	+	+	+	+	+
pSS23[a]	40	+	+	+	+	+	+
pSS24[a]	47	+	+	+	+	+	+
pSS25[a]	64	+	+	+	+	+	+
pSS26[a]	39	+	+	+	+	+	+
pSS27[a]	51	+	+	+	+	+	+

[a]Patients with pSS included in pooled tear sample only. [b]Autoantibody production was assessed by ELISA. [c]Values are in mm/5 minutes; normal flow >5 mm/5 minutes. The + symbol indicates dryness and tear secretion <5 mm/5 minutes. [d]Values are in ml/15 minutes; normal flow >1.5 ml/15. The + symbol indicates dryness and stimulated whole saliva secretion <3.5 ml/5 minutes

Saliva and tear fluid collection

Saliva collection at the Dry Mouth Clinic

Participants underwent a thorough oral examination at the Dry Mouth Clinic, and stimulated whole saliva was collected from all participants. Subjects were asked to not have any food or drink for at least 1 hour before saliva collection. Following the oral examination, the participants were asked to chew on a paraffin block (Paraffin Pellets, Ivoclor Vivadent, Shaen, Lichtenstein), while saliva was collected on ice for 5 minutes between 9.00 a.m. and 3.00 p.m. As secretory ability has been shown to vary depending on stimulation by chewing, and on the time of day, these strict routines were employed to ensure standardisation of the method for saliva collection. The samples were weighed to determine volume, where only patients producing ≥800 μl of stimulated whole saliva were included in the study. All samples were then aliquoted and stored at -80 °C.

Tear fluid collection at the Norwegian Dry Eye Clinic

Participants underwent a thorough ocular surface examination at the Norwegian Dry Eye Clinic. Tear fluid was collected from both eyes by placing a Schirmer tear test strip (HAAG-STREIT, Essex, UK) on each eye for 5 minutes, or more to produce a minimum combined total of 10 mm of tear volume from both eyes at room temperature. Each Schirmer strip was then transferred to 500 μl of 0.1 μm filtered phosphate-buffered saline (PBS) (Gibco, pH 7.4, ThermoFisher Scientific, Oslo, Norway) and stored at -80 °C.

Extraction of EVs from saliva

EVs were isolated from stimulated whole saliva using size-exclusion chromatography, as described previously [44]. In brief, the saliva samples were centrifuged at 300 rpm for 10 minutes to remove debris, and then diluted 1:2 with 0.1 μm filtered PBS. A qEV size-exclusion chromatography column (iZON Science, Oxford, UK) was equilibrated by washing the column with 15 ml of 0.1 μm filtered PBS; 1 ml of the diluted saliva was then applied to the column and 16 fractions, each 500 μl in volume, were collected by continuously adding 0.1 μm filtered PBS to the column. To standardise the procedure, elution time frames were recorded when reaching fractions 7, 12 and 15, and the number of eluted drops in fraction 10 was also recorded. A new column was used for each saliva sample. The eluted fractions 8 – 10 (containing the majority of microvesicles and exosomes

present in the samples) were concentrated for 80 minutes at 30 °C in a MiVac centrifugal vacuum concentrator (SP Scientific, Suffolk, UK) from a volume of 500 μl to approximately 250 μl. Fractions 8–10 were collected into a joint fraction and the protein concentration was determined using Qubit Fluorometric Quantitation (Thermo-Fisher Scientific, Oslo, Norway). A volume of the diluted stimulated whole saliva (100 μl) and the joint fractions from each participant were then sent for proteomic analysis while preserved on dry ice.

Extraction of EVs from tear fluid

For each subject, tear fluid eluted from Schirmer strips into 0.1 μm filtered PBS (1 ml; pooling of 500 μl PBS containing a Schirmer strip from each eye) was applied to an equilibrated qEV size exclusion chromatography column. Fractions of 500 μl were eluted and concentrated, and fractions 8–10 were collected into a joint fraction and the protein concentration was determined as described above. A new column was used for each sample. Due to the low numbers of proteins and vesicles in tear fluid collected from the individual patients with pSS (minimum 10 mm fluid per patient), tear fluid from Schirmer strips containing 80 mm tear fluid from 11 patients with pSS was pooled in 5 ml PBS. The pooled sample was subsequently concentrated to 200 μl using Amicon Ultra-4 columns and furthermore adjusted to a volume 1.0 ml with PBS before being applied on a qEV column. Schirmer strips also containing 80 mm tear fluid from five controls were handled in parallel. These pooled tear fluid samples were included for verification. A small volume from the tear fluid sample of each participant (100 μl), the joint fractions from each individual, and the joint fractions from pooled tear samples of the patients with pSS and the controls were then sent for proteomic analysis while preserved on dry ice.

Characterisation of EVs

Nanoparticle tracking analysis

Nanoparticle tracking analysis was conducted on joint fractions from saliva and tear fluid to determine size distribution and concentration of the respective EVs using a NanoSight NS500 instrument (Malvern Instruments Ltd, Malvern, UK), equipped with a scientific cMOS camera with trigger, a 488-nm laser, and a syringe pump for continuous sample flow. Samples were diluted in 0.02 μm filtered PBS to reach the measurement range (10^8 – 10^9 particles/ml). Analysis was performed using the NTA 3.0 software (Malvern Instruments, Malvern, UK). Briefly, a video capture of 60 seconds per sample was applied. The camera level was set to 14–15 for saliva and 12–15 for tear fractions, and the detection threshold was set to 3. The hydrodynamic diameter of the particles in each sample was calculated by the software, through registering their Brownian motion in response to laser light scattering, utilising the Stokes Einstein equation. Sample concentration was estimated as a subsequent parameter of the sample volume. A summary of the measurements obtained from the nanoparticle tracking analysis for EV characterisation in saliva and tear fluid is presented in Table 2.

Flow cytometry detection of CD9 positive EVs

Immunoaffinity capture and detection of CD9 positive EVs from joint fractions was performed using the Exosome Human CD9 Flow Detection Kit (Dynal®, Thermo-Fisher Scientific, Oslo, Norway) and flow cytometry. In brief, 100 μl of each joint fraction was incubated overnight with prewashed 20 μl Dynabeads (2.7 mm) on a HulaMixer Sample mixer at 4 °C. The bead-captured EVs were then washed three times with 0.1 μm filtered PBS containing 0.1% bovine serum albumin (BSA). Subsequently, they were incubated with RPE-conjugated detection antibody (anti-human CD9-RPE clone ML-13, BD Biosciences, Oslo, Norway), or isotype control (IgG1-RPE, BD Biosciences, Oslo, Norway), for 45 minutes at room temperature on an orbital shaker (1000 rpm), protected from light. The bead-containing samples were further washed twice with PBS containing 0.1% BSA before proceeding with flow cytometry analysis, using a BD Accuri™ C6 Cytometer (BD Biosciences, Oslo, Norway). Median fluorescence intensity (MFI) was reported as a signal to noise (S/N) ratio to isotype control from a total of 300 singlet events. Measurements obtained from the flow cytometry analyses

Table 2 Characterisation of EVs in saliva and tear fluid

	Mean particle size[a](nm)	Particles/ml[a]	CD9+ EVs S/N ratio MFI[b]
Saliva			
Patients with pSS	189 ± 4.1	5.46 E + 10 ± 1.43 E + 10*	3.47 ± 0.56*
Controls	189 ± 4.4	2.41 E + 10 ± 3.98 E + 09	1.93 ± 0.15
Tear fluid			
Patients with pSS	171 ± 6.9	1.54 E + 09 ± 3.08 E + 08	1.10 ± 0.03
Controls	163 ± 9.6	1.09 E + 09 ± 1.06 E + 08	1.06 ± 0.02
Pool of patients with pSS	190	2.04 E + 10	2.88
Pool of controls	144	8.45 E + 09	1.06

[a]Nanoparticle tracking analysis was conducted on extracellular vesicles (EV) joint fractions from whole saliva (n = 19 patients with primary Sjögren's syndrome (pSS), n = 32 controls), tear fluid (n = 7 patients with pSS, n = 6 controls), and one pooled tear sample (n = 11 patients with pSS, n = 5 controls) to determine mean particle size of microvesicles and exosomes (nm ± SEM), in addition to concentrations of EVs (particles/ml ± SEM).[b]Detection of CD9+ EVs from joint fractions of saliva (n = 19 patients with pSS, n = 32 controls), tear fluid (n = 11 patients with pSS, n = 10 controls), and one pooled tear sample (n = 11 patients with pSS, n = 5 controls) was performed by immunoaffinity capture using anti-CD9-coated magnetic beads followed by flow cytometry analysis. The results were reported as signal-to-noise (S/N) ratios of median fluorescence intensity (MFI). *Significant difference between patients with pSS and controls (unpaired *t* test, $p < 0.05$)

for EV characterisation in saliva and tear fluid are presented in Table 2.

Determination of protein amount

Proteomics analysis was executed on saliva and tear fluid from both patients with pSS and controls before and after isolation of EVs. Total protein concentration (mg/ml) in the saliva samples ranged from 0.5 to 1.36 in patients with pSS, and from 0.25 to 0.94 in controls. Meanwhile, saliva joint fractions showed a total protein range of 0.04 to 0.07 in patients with pSS, and 0.02 to 0.07 in controls. The total protein concentration in tear samples ranged from 0.27 to 0.70 in patients with pSS, while in controls this ranged from 0.22 to 0.70. Tear joint fractions displayed a total protein range from 0.03 to 0.05 in patients with pSS, and 0.03 to 0.04 in controls. The total protein in the pooled tear sample was 0.47 mg/ml in the patients with pSS and 0.35 mg/ml in the controls. Additionally, the joint fractions in the pooled tear sample exhibited a total protein value of 0.03 mg/ml in the patients with pSS, and 0.03 mg/ml in the controls.

In-solution protein digestion

For saliva and EVs of saliva, four times the sample volume of ice-cold acetone was added to each sample, vortexed and precipitated overnight at -20 °C. Samples were then centrifuged at 16,000 g for 20 minutes at 4 °C (Centrifuge 5415R, Eppendorf, Hamburg, Germany) and the supernatants were discarded. Proteins were re-dissolved in 50 μl of a mixture of 6 M urea and 100 mM ammonium bicarbonate (pH 7.8). For reduction and alkylation of cysteines, 2.5 μl of 200 mM DTT in 100 mM Tris-HCl (pH 8) was added and the samples were incubated at 37 °C for 1 hour followed by the addition of 7.5 μl of 200 mM iodoacetamide for 1 hour at room temperature in the dark. The alkylation reaction was quenched by adding 10 μl of 200 mM DTT at 37 °C for 1 hour. For all samples, the proteins were digested with 10 μg of trypsin for 16 hours at 37 °C. The digestion was stopped by adding 5 μl of 50% formic acid. The generated peptides were purified using an OMIX C18-micro SPE (Agilent, Santa Clara, CA, USA), and then dried using a Speed Vac concentrator (Concentrator Plus, Eppendorf, Hamburg, Germany).

Liquid LC-MS

The tryptic peptides were dissolved in 10 μl of 0.1% formic acid/2% acetonitrile, and 5 mμl was analysed using an Ultimate 3000 RSLCnano-UHPLC system connected to a Q Exactive mass spectrometer (Thermo Fisher Scientific, Bremen, Germany), and also equipped with a nano electrospray ion source. For liquid chromatography separation, an Acclaim PepMap 100 column was used (C18, 2 μm beads, 100 Å, 75 μm inner diameter, 50 cm length) (Dionex, Sunnyvale CA, USA). A flow rate of 300 nl/minute was employed with a solvent gradient of 4 – 35% B in 47 minutes, to 50% B in 3 minutes and then to 80% B in 2 minutes. Solvent A was 0.1% formic acid and solvent B was 0.1% formic acid/90% acetonitrile.

The mass spectrometer was operated in the data-dependent mode to automatically switch between MS and MS/MS acquisition. Survey full-scan MS spectra (from m/z 300 to 2,000) were acquired with the resolution R = 70 000 at m/z 200, after accumulation to a target of 1e6. The maximum allowed ion accumulation time was 60 milliseconds. The method used allowed sequential isolation of up to the ten most intense ions, depending on signal intensity (intensity threshold 1.7e4), for fragmentation using higher-energy collisional-induced dissociation (HCD) at a target value of 10,000 charges, and a resolution R = 17,500. Target ions already selected for MS/MS were dynamically excluded for 60 seconds. The isolation window was m/z = 2 without offset. For accurate mass measurements, the lock mass option was enabled in MS mode.

Data were acquired using Xcalibur v2.5.5 and raw files were processed to generate peak list in Mascot generic format (*.mgf) using ProteoWizard release version 3.0.331. Database searches were performed using Mascot in-house version 2.4.0 to search the SwissProt database (Human, 20,279 proteins) assuming the digestion enzyme trypsin, at maximum of one missed cleavage site, fragment ion mass tolerance of 0.05 Da, parent ion tolerance of 10 ppm, and oxidation of methionines, and acetylation of the protein N-terminus as variable modifications. For saliva and EVs of saliva, carbamidomethylation of cysteines as fixed modification was used in addition.

Data processing and statistical analysis

Scaffold (version Scaffold_4.4, Proteome Software Inc., Portland, OR, USA) was used to validate MS/MS-based peptide and protein identifications. Peptide identifications were accepted if they could be established at greater than 95.0% probability by the Scaffold Local false discovery rate (FDR) algorithm. Protein identifications were accepted if they could be established at greater than 99.0% probability. For label-free quantification, the entire MS2 total ion current (TIC) across all biological replicates was evaluated using the t test. For functional analysis of the proteomics data, the Search Tool for the Retrieval of Interacting Genes/Proteins) (STRING) (http://string-db.org/) and the Database for Annotation, Visualization and Integrated Discovery (DAVID) (v 6.7, https://david.ncifcrf.gov) were applied. Stimulated whole saliva, saliva EVs (joint fractions) and tear fluid were analysed individually, comparing the 10 patients with pSS and controls with the highest number of proteins. STRING was used to explore how these proteins were interrelated to form protein-protein interaction networks, by applying all active interaction sources (experiments, databases and text mining), and medium confidence. Furthermore, DAVID was applied, using an FDR with a maximum

5% cutoff, in order to delineate specific cellular pathways involving these upregulated proteins in the patients with pSS. The unregulated group of proteins was also examined and compared to the DAVID analysis for each of stimulated whole saliva, saliva EVs (joint fractions) and tear samples.

Results

Workflow for the identification of proteins upregulated in patients with pSS

The proteome of saliva, tear fluid, and EVs of both saliva and tear fluid from patients and controls were examined by digestion of the proteins with trypsin, analysis of the proteins by LC-MS, identification of the proteins using Mascot database searches and further data analysis using Scaffold to find quantitative differences based on the *t* test applied on MS2 total ion currents. Significantly upregulated proteins with *p* values <0.05 according to the *t* test were further analysed using DAVID for gene ontology (GO) term overrepresentation and STRING for protein-protein network analysis.

Upregulation of proteins involved in innate immunity, cell signalling and wound repair in whole saliva from patients with pSS

LC-MS analysis of whole saliva from 20 patients and 32 healthy controls identified approximately 500 unique proteins with 48,424 peptide spectrum matches. Thirty-eight proteins were upregulated in whole saliva in the pSS patient group compared to the controls (Scaffold: t test, $p < 0.05$) (Additional file 1: Table S1).

GO overrepresentation analysis using DAVID indicated that cellular pathways for the upregulated proteins in the whole saliva from the pSS patient group, in comparison to unregulated proteins, included lymphocyte-mediated immunity, calcium ion binding and the neutrophin signalling pathway. These pathways are all components of the adaptive immune response (Fig. 1).

The STRING analysis revealed that the upregulated proteins in whole saliva from patients with pSS formed two distinct protein-protein interaction networks; one is involved in metabolism and redox reactions, while the other plays a central role in both innate and adaptive immunity (Fig. 2). Assuming proteins found only in the pSS patient group would be the most promising candidates for potential disease biomarkers, we also considered the number of biological replicates in our analyses. Accordingly, the five most deviating upregulated proteins considering biological replicates and spectral counts were neutrophil gelatinase-associated lipocalin (LCN2), granulins (GRN), calmodulin (CALM), epididymal secretory protein 1 (NPC2), and calmodulin-like protein 5 (CALML5), in descending order (Table 3, Additional file 2: Figure S1). The most upregulated protein in whole saliva from patients with pSS, namely LCN2, is an iron-binding protein involved in apoptosis and the innate immune system, and is particularly responsible for the activation of neutrophils. It is also an indicator of acute renal failure. LCN2 is therefore present within the protein network involved in both innate and adaptive immunity (Fig. 2). GRN is a cytokine-like peptide that is central in inflammation due to its active role in wound repair and tissue remodelling. CALM and CALML5 are calcium-binding proteins and play a role in intracellular signalling and differentiation of keratinocytes, respectively. ESP1 is a cholesterol transporter involved in cholesterol homeostasis within the endosome and/or lysosome (Table 3).

EVs in whole saliva from patients with pSS express abundant proteins vital for activation of the innate immune system and adipocyte differentiation

LC-MS analysis of EVs from whole saliva from 20 patients and 32 healthy controls identified around 500 unique proteins with 48,620 peptide spectrum matches. Thirty six proteins were significantly upregulated in patients with pSS compared to controls (Scaffold, *t* test, $p < 0.05$) (Additional file 1: Table S2).

The DAVID analysis of EVs from whole saliva revealed cellular pathways involved in adhesion, cytoskeleton organisation and membrane fusion. Together, these pathways are involved in cellular component assembly, and possess the most significantly changed GO terms when compared with the identified unregulated proteins (Fig. 1).

One major protein-protein interacting network was identified for the upregulated proteins in EVs isolated from whole saliva from patients with pSS. This observation was visualised using STRING analysis (Fig. 3). These proteins are active players in the cytoskeleton, and are also involved in cell migration and cell junction. The five upregulated proteins that deviated most in biological replicates between patients with pSS and controls and that were detected in EVs from whole saliva were adipocyte plasma membrane-associated protein (APMAP), guanine nucleotide-binding protein subunit alpha-13 (GNA13), WD repeat-containing protein 1 (WDR1), tyrosine-protein phosphatase non-receptor type substrate 1 (SIRPA), and lymphocyte-specific protein 1 (LSP1) (Table 4, Additional file 3: Figure S2).

The most changed of these proteins in EVs from whole saliva, APMAP, is an enzyme central in adipocyte differentiation. Moreover, GNA13 is a G-protein that consequently plays a role in transmembrane signalling, while WDR1 is a regulatory protein involved in the disassembly of actin filaments. Interestingly, SIRPA is a glycoprotein present in innate immunity, particularly in the regulation of NK cells and dendritic cell inhibition. LSP1 is an actin-binding protein also involved in innate immunity, specifically neutrophil activation, and chemotaxis (Table 4). Out of the five most upregulated proteins; both GNA13 and WDR1 are present within the protein network identified (Fig. 3).

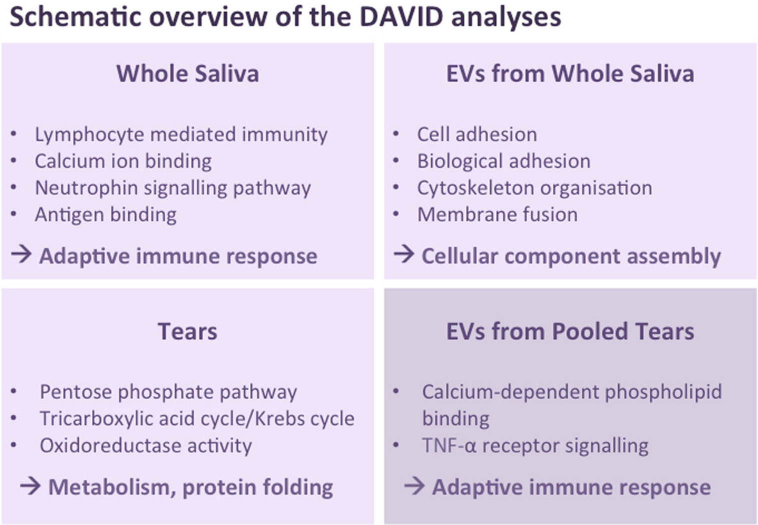

Fig. 1 Database for Annotation, Visualization and Integrated Discovery (*DAVID*) analysis delineating cellular pathways that involve proteins identified in whole saliva, tear fluid, and extracellular vesicles (*EVs*). Cellular pathways involving innate and adaptive immune responses, cellular component assembly, metabolism and protein folding were identified using DAVID (v 6.7, https://david.ncifcrf.gov) analysis for each sample of whole saliva, tear fluid and EVs

Overexpression of proteins involved in TNF-α signalling and B cell survival detected in tear fluid from patients with pSS

Tear fluid from 11 patients and 11 healthy controls was analysed using LC-MS, and more than 900 unique proteins were identified with 75,701 peptide spectrum matches. The application of MS2 TICs using Scaffold, following proteomic analysis, allowed the identification of 197 significantly upregulated proteins in tear fluid from the patient group (Additional file 1: Table S3).

DAVID revealed cellular pathways distinguished from upregulated proteins in tear fluid from patients with pSS, which entail the Pentose phosphate pathway, the tricarboxylic acid/Krebs cycle, and oxidoreductase activity, which are all elements of metabolism and protein folding (Fig. 1).

By applying STRING analysis we were able to visualise two protein-protein interaction networks encompassing the upregulated proteins in tear fluid from patients with pSS; one is involved in redox-reactions and oxidative stress, while the other protein-protein network is central in the formation of the cytoskeleton and cell migration (Fig. 4). The five most upregulated proteins present in tear fluid from patients with pSS were DNA (apurinic or apyrimidinic site) lyase (APEX1), thioredoxin-dependent peroxidase reductase (PRDX3), copine (CPNE1), aconitate hydratase (ACO2), and LIM domain only protein 7 (LMO7) (Table 5, Additional file 4: Figure S3). Interestingly, APEX1 is an enzyme that is activated in response to oxidative stress, and is involved in DNA repair and the regulation of transcriptional factors. PRDX3 is an enzyme that regulates NF-kappa-B activation, and thereby plays a central role in B cell survival. CPNE1 is a calcium-dependent phospholipid-binding protein involved in TNF-α receptor signalling, and in turn in inflammation and apoptosis. ACO2 is an enzyme of the Krebs cycle with a key role in carbohydrate metabolism. Finally, LMO7 is described as a multifunctional protein with a central role in cell signalling, cell adhesion, and ubiquitination (Table 5). Of the five most deviating upregulated proteins in patients with pSS, PRDX3 is present within the protein network involving redox-reactions and oxidative stress (Fig. 4).

Additionally, a combined analysis of protein changes in both stimulated whole saliva and tear fluid was performed using Scaffold in the pSS patient group and the controls. Out of all the aforementioned proteins in the study, LCN2 was found to be upregulated in both stimulated whole saliva and tear fluid in patients with pSS.

Low tear fluid volumes were collected individually from each patient with pSS, leading to fewer than 100 proteins identified in most of the samples in which EVs were isolated individually from the tear fluid of each participant. However, on proteomic analysis of EVs extracted from the pooled tear sample combined from 11 patients with pSS, CPNE1 and CALM were expressed more in the patient group. Moreover, the DAVID analysis of the pooled tear sample revealed cellular pathways involved in calcium-dependent phospholipid binding, and TNF-α receptor signalling, both of which are components of the adaptive immune response and comparable to the calcium ion binding pathways identified in whole saliva (Fig. 1).

Discussion

By studying the proteome of biological fluids through LC-MS approaches, and possibly combined with the

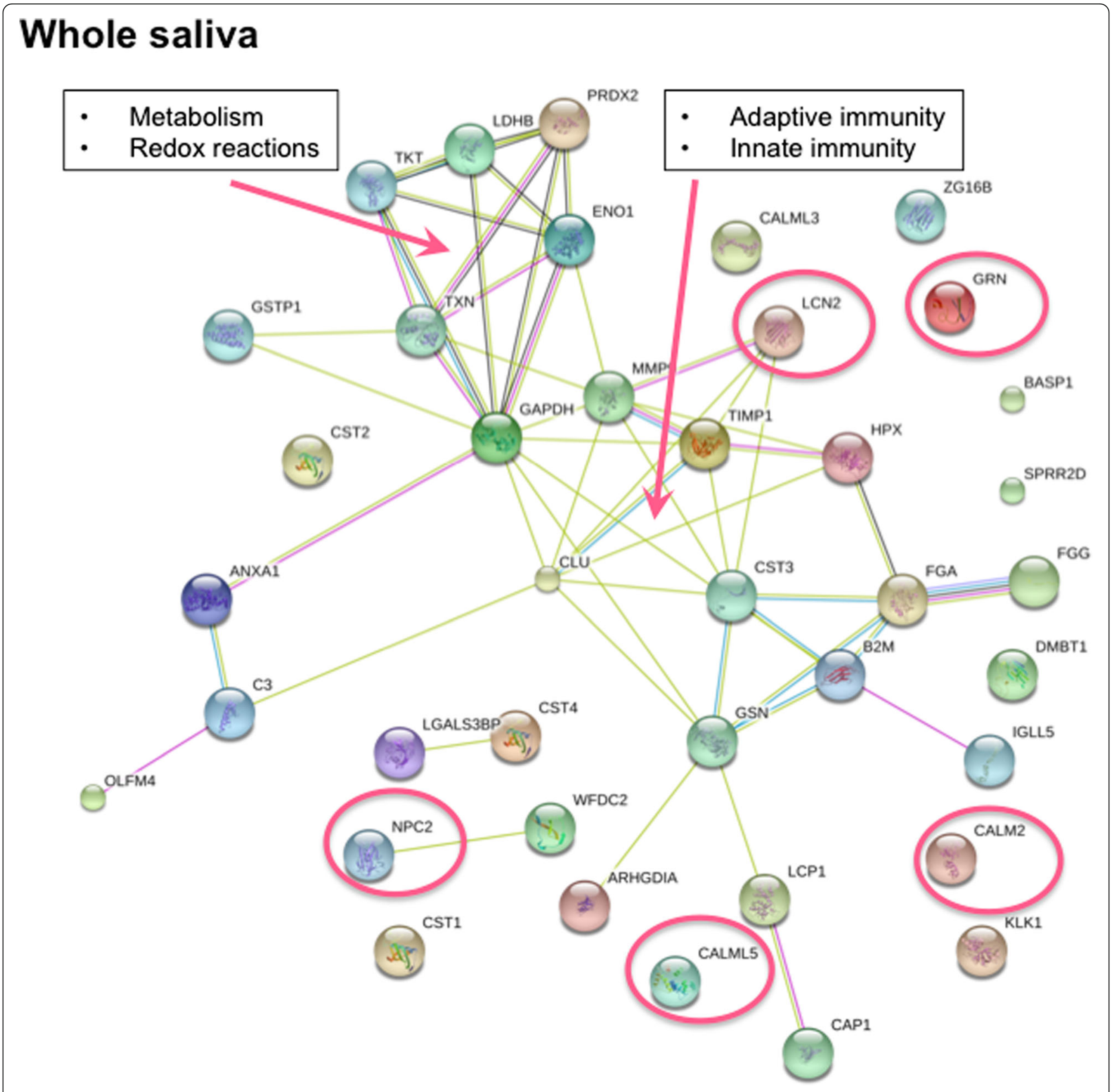

Fig. 2 Protein-protein interaction networks of upregulated proteins associated with primary Sjögren's syndrome identified in stimulated whole saliva. Two distinct protein-protein interaction networks are visualised. One is involved in metabolism and redox reactions, while the other plays a central role in both innate and adaptive immunity and contains the most upregulated protein in the patient group, namely neutrophil gelatinase-associated lipocalin (*LCN2*). The five most upregulated proteins in the patient group (Table 3) are indicated with *red circles*. The Search Tool for the Retrieval of Interacting Genes/Proteins (http://string-db.org/) was used to generate the networks, where potential interactions of proteins with medium confidence are shown. The different clusters are indicated by the same colour. The colour of the *connecting lines* indicates the type of evidence used in predicting the associations (*red* gene fusion, *yellow* text-mining extracted from literature, *purple* protein-protein interaction datasets, *light blue* protein interaction groups, *black* linked across species). *CALM* calmodulin, *CALML5* calmodulin-like protein 5, *GRN* granulin adipocyte plasma, *APMAP* membrane-associated protein, *GNA13* guanine nucleotide-binding protein subunit alpha-13, *WDR1* WD repeat-containing protein 1, *SIRPA* tyrosine-protein phosphatase non-receptor type substrate 1, *LSP1* lymphocyte-specific protein 1

chromatographic separation of extracellular vesicles from other biomolecules, the search for potential biomarkers and therapeutic targets can be realised for SS. Such biomarkers can in turn be implemented as potential non-invasive diagnostic tools that can be applied for monitoring disease progression. The majority of proteomic studies of SS have been based on saliva as the biological fluid, using different mass spectrometry approaches, in addition

Table 3 Highly upregulated proteins in stimulated whole saliva from patients with pSS

Number	Gene	Related protein[a]	Replicates (pSS : C)	Spectral counts (pSS : C)	Classification and function[b]
1	LCN2	Neutrophil gelatinase-associated lipocalin	10 : 2	38 : 2	Iron-binding protein; innate immunity (neutrophils)
2	GRN	Granulins	7 : 0	12 : 0	Cytokine-like peptide; inflammation, wound repair, tissue remodelling
3	CALM	Calmodulin	7 : 1	17 : 1	Calcium-binding protein; intracellular signalling
4	NPC2	Epididymal secretory protein 1	6 : 0	11 : 0	Cholesterol transporter; cholesterol homeostasis (endosome/lysosome)
5	CALML5	Calmodulin-like protein 5	5 : 0	13 : 0	Calcium-binding protein; differentiation of keratinocytes

[a]The five most upregulated proteins in whole saliva from patients with primary Sjögren's syndrome (pSS) deviating in replicates, i.e. number of individuals (frequency), and spectral counts, as identified by proteomics analysis and Scaffold (v 4.4.6, http://www.proteomesoftware.com/products/scaffold/). [b]The classification and functions of the proteins presented were identified using publicly available databases, such as UniProt (http://www.uniprot.org). *C* controls

to genomics [14, 16–21]. This confirms that saliva not only reflects the salivary gland involvement that characterises the disease process in SS [18, 24, 25], but additionally has the potential to communicate an individual's current health [26, 27].

So far tear fluid has only been used to identify potential biomarkers for pSS in a limited number of proteomic studies [22, 23]. Hence, in the current study we hypothesised that by applying LC-MS alone, and in combination with size-exclusion chromatography of both stimulated whole saliva and tear fluid collected from patients with pSS and from healthy controls, novel biomarkers encompassing both salivary and lacrimal disease target organs could possibly be identified.

In order to delineate cellular pathways involving the upregulated proteins identified with LC-MS in the samples from the patients with pSS, GO and Kyoto Encyclopedia of Genes and Genomes (KEGG) pathway overrepresentation analyses using DAVID were performed. Our results demonstrated pathways of the adaptive immune response in the whole saliva, of the cellular component assembly in the EVs extracted from whole saliva, of metabolism and protein folding in the tear fluid of patients with pSS and finally, components of the adaptive immune response in the EVs isolated from the pooled sample of tear fluid from patients with pSS, which was comparable to the calcium ion binding pathways identified in whole saliva (Fig. 1). Viewed as a whole, the identified cellular pathways and components clearly indicate the involvement of autoimmune reactions and over-activation of the adaptive and innate immune systems in patients with pSS, both as a consequence of disease pathogenesis and probably also as part of the healing process.

The LC-MS analyses indicate upregulation of proteins involved in innate immunity, cell signalling and wound repair in whole saliva from patients with pSS. Interestingly, LCN2, the most upregulated protein in whole saliva from patients with pSS, is an iron-binding protein involved in the innate immune system, and is particularly responsible for the activation of neutrophils [50]. This suggests the involvement of viral infection in SS pathogenesis. A similar implication was depicted by Hu and co-workers [18], where they identified salivary proteomic and genomic biomarkers for SS showing upregulation of genes involved in the IFN pathway, thereby suggesting a potential role for viral infection in SS. Moreover, both GRN, a cytokine-like peptide that is central in inflammation due to its active role in wound repair and tissue remodelling [51], and CALML5, a calcium-binding protein that plays a central role in the differentiation of keratinocytes [52], were also upregulated in our patient group. This finding in turn provides evidence of acinar damage and oral environment alteration.

Both Giusti et al. [13] and Fleissig et al. [19] identified biomarkers that might include specific indication of tissue damage (e.g. actin), inflammation (e.g. calgranulins), and tissue repair (e.g. keratin 6 L) in unstimulated whole saliva. The present study identified similar potential with GRN and CALML5 in stimulated whole saliva (Table 3, Additional file 2: Figure S1). Furthermore, CALM, a calcium-binding protein that plays a role in intracellular signalling, and ESP1, a cholesterol transporter involved in cholesterol homeostasis within the endosome and/or lysosome, were also upregulated in whole saliva from patients with pSS. Similarly, previous proteomic studies on whole saliva have determined broad and distinct protein patterns that are characteristic of SS, including secretory proteins, enzymes, highly abundant immune system-related molecules (e.g. β2-microglobulin), and cytokines such as IL-4 and IL-5 [21, 28, 29]. The current STRING analysis of whole saliva also strengthens the concept of involvement and over-activation of the innate and adaptive immune system in SS. This is presumably due to the upregulation of LCN2 and other related pro-inflammatory-related proteins in the patients with pSS to form protein-protein network interactions (Fig. 2).

Using size exclusion chromatography on whole saliva to isolate EVs followed by LC-MS allowed us to identify potential biomarkers that are vital for activation of the innate immune system and adipocyte differentiation. More precisely,

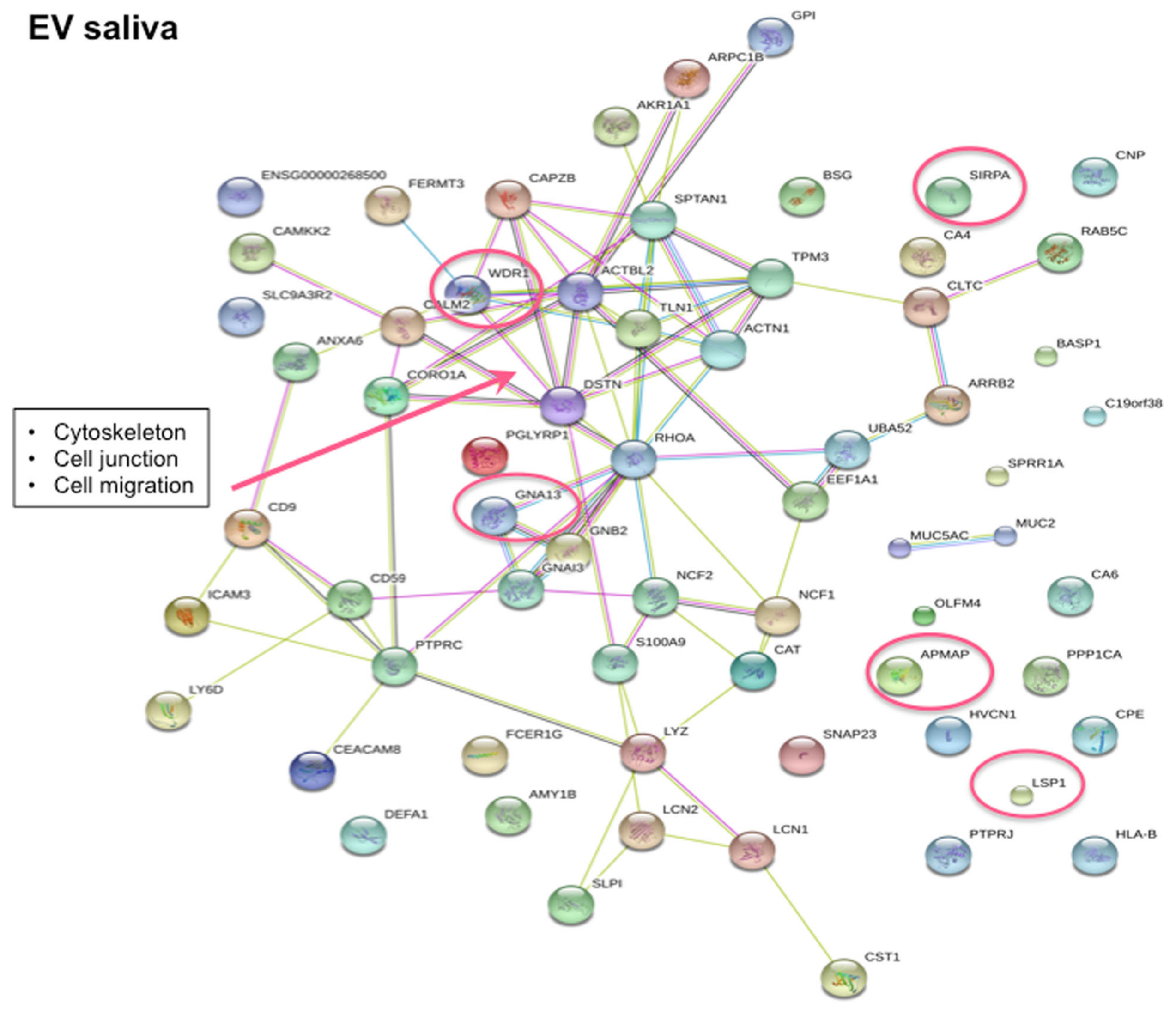

Fig. 3 Protein-protein interaction networks of upregulated proteins associated with primary Sjögren's syndrome detected in extracellular vesicles from whole saliva. One major protein-protein interaction network is visualised. The proteins identified are involved in the cytoskeleton, in addition to cell migration and cell junction. Out of the five most upregulated proteins in pSS (Table 4), indicated with *red circles*, both guanine nucleotide-binding protein subunit alpha-13 (*GNA13*) and WD repeat-containing protein 1 (*WDR1*) are present within this protein network. The Search Tool for the Retrieval of Interacting Genes/Proteins (http://string-db.org/) was used to generate the networks, where potential interactions of proteins with medium confidence are shown. The different clusters are indicated by the same colour. The colour of the *connecting lines* indicates the type of evidence used in predicting the associations (*red* gene fusion, *yellow* text-mining extracted from literature, *purple* protein-protein interaction datasets, *light blue* protein interaction groups, *black* linked across species). *APMAP* membrane-associated protein, *SIRPA* tyrosine-protein phosphatase non-receptor type substrate 1, *LSP1* lymphocyte-specific protein 1

the most upregulated protein in EVs from whole saliva, namely APMAP, is an enzyme central to adipocyte development. We have recently shown increased occurrence of adipose tissue replacement in minor salivary gland biopsies from patients with SS [53]. Interestingly, these adipocytes were detected in areas rich in IL-6, suggesting their active involvement in immune reactions. Hence, the upregulation of APMAP in stimulated whole saliva could be an indication of adipocyte involvement in disease progression. Moreover, both GNA13, a G-protein that consequently plays a role in transmembrane signalling, and WDR1, a regulatory protein involved in the disassembly of actin filaments, are proteins needed to drive inflammation and tissue damage, respectively.

Interestingly, SIRPA is another potential biomarker of the pro-inflammatory process, as it regulates NK cells and dendritic cells. Furthermore, LSP1, being involved in innate immunity, specifically neutrophil activation and chemotaxis, is another possible indicator of the involvement of viral infection in the pathogenesis of SS (Table 4, Additional file 3: Figure S2). One major protein-protein interacting network was identified for EVs isolated from

Table 4 Highly upregulated proteins in EVs isolated from stimulated whole saliva from patients with pSS

Number	Gene	Related protein[a]	Replicates (pSS : C)	Spectral counts (pSS : C)	Classification and function[b]
1	APMAP	Adipocyte plasma membrane-associated protein	7 : 0	26 : 0	Enzyme; adipocyte differentiation
2	GNA13	Guanine nucleotide-binding protein subunit alpha-13	6 : 0	11 : 0	G-protein; transmembrane signalling
3	WDR1	WD repeat-containing protein 1	6 : 0	11 : 0	Regulatory protein; disassembly of actin filaments
4	SIRPA	Tyrosine-protein phosphatase non-receptor type substrate 1 (PTPN1)	6 : 0	9 : 0	Glycoprotein; innate immunity (NK cells), dendritic cell inhibition
5	LSP1	Lymphocyte-specific protein 1	5 : 0	9 : 0	Actin-binding protein; neutrophil activation, chemotaxis

[a]The five most upregulated proteins in extracellular vesicles isolated from whole saliva from patients with primary Sjögren's syndrome (pSS) deviating in replicates, i.e. number of individuals (frequency), and spectral counts, as identified by proteomics analysis and Scaffold (v 4.4.6, http://www.proteomesoftware.com/products/scaffold/). [b]Classification and functions of the proteins presented were identified using publicly available databases, such as UniProt (http://www.uniprot.org). *C* controls, *NK* natural killer

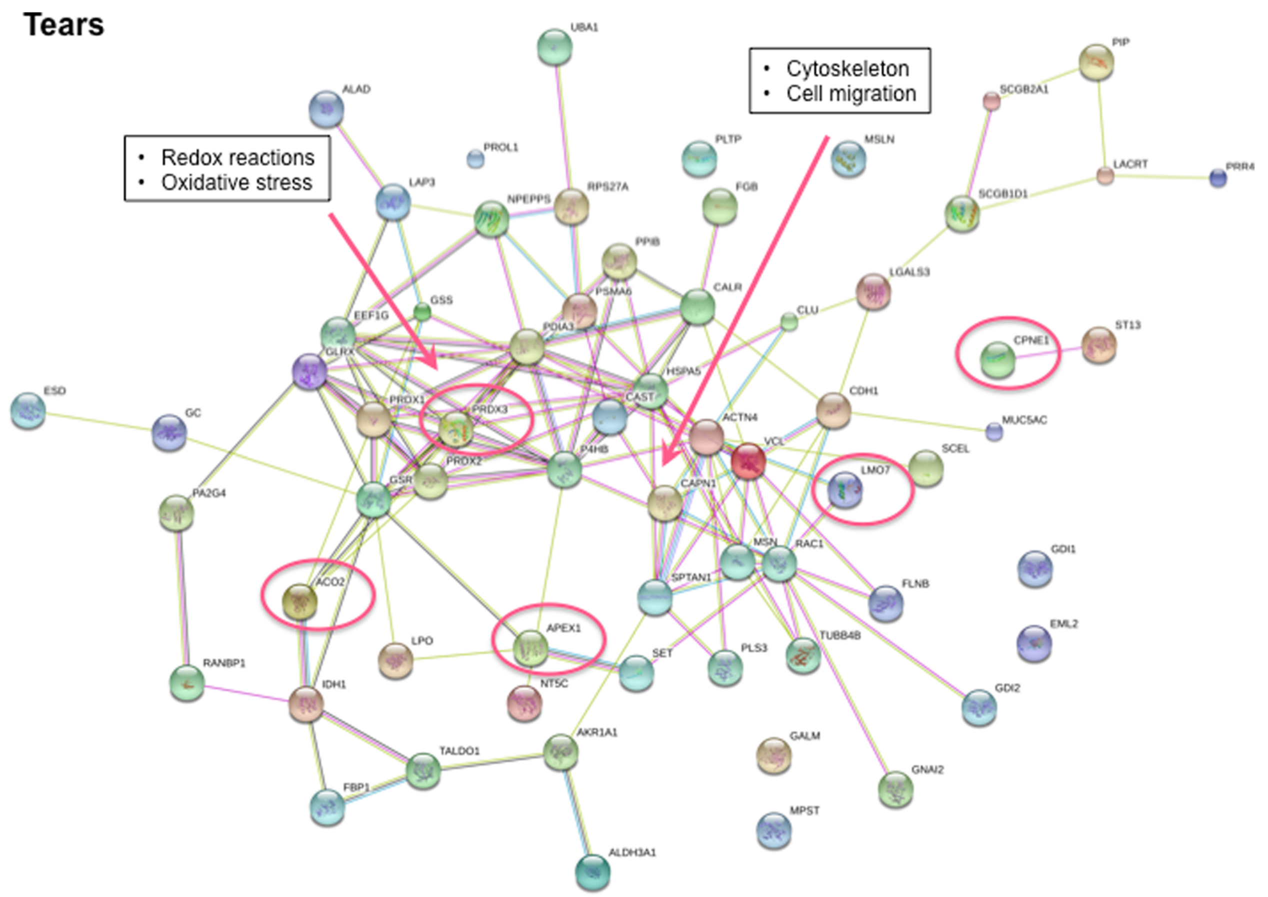

Fig. 4 Protein-protein interaction networks of upregulated proteins associated with primary Sjögren's syndrome detected in tear fluid. Two protein-protein interaction networks are visualised. One is central in the formation of the cytoskeleton and cell migration, while the other is involved in redox reactions and oxidative stress and contains one out of the five most upregulated proteins, as indicated with *red circles* (Table 5), namely thioredoxin-dependent peroxidase reductase (*PRDX3*). The Search Tool for the Retrieval of Interacting Genes/Proteins (http://string-db.org/) was used to generate the networks, where potential interactions of proteins with medium confidence are shown. The different clusters are indicated by the same colour. The colour of the *connecting lines* indicates the type of evidence used in predicting the associations (*red* gene fusion, *yellow* text-mining extracted from literature, *purple* protein-protein interaction datasets, *light blue* protein interaction groups, *black* linked across species). *APEX1* DNA (apurinic or apyrimidinic site) lyase, *CPNE1* copine, *ACO2* aconitate hydratase, *LMO7* LIM domain only protein 7

Table 5 Highly upregulated proteins in tear fluid from patients with pSS

Number	Gene	Related protein[a]	Replicates (pSS : C)	Spectral counts (pSS : C)	Classification and function[b]
1	APEX1	DNA-(apurinic or apyrimidinic site) lyase	10 : 1	22 : 1	Enzyme; oxidative stress, DNA repair, regulation of transcriptional factors
2	PRDX3	Thioredoxin-dependent peroxidase reductase	8 : 0	15 : 0	Enzyme; redox regulation, regulates NF-kappa-B activation (B cell survival)
3	CPNE1	Copine 1	9 : 2	23 : 2	Phospholipid-binding protein (calcium-dependent), TNF-α receptor signalling
4	ACO2	Aconitate hydratase	7 :0	19 : 0	Enzyme; tricarboxylic acid cycle/Krebs cycle, carbohydrate metabolism
5	LMO7	LIM domain only protein 7	7 : 0	9 : 0	Cell signalling, cell adhesion, ubiquitination

[a]The five most upregulated proteins in tear fluid from patients with primary Sjögren's syndrome (pSS) deviating in replicates, i.e. number of individuals (frequency), and spectral counts, as identified by proteomics analysis and Scaffold (v 4.4.6, http://www.proteomesoftware.com/products/scaffold/). [b]Classification and functions of the proteins presented were identified using publicly available databases, such as UniProt (http://www.uniprot.org) *C* controls

whole saliva, including proteins that regulate the formation and disassembly of the cytoskeleton, in addition to their involvement in cell migration and cell junction. Both GNA13 and WDR1 were present within this protein network (Fig. 3).

As only a limited number of proteomic studies have so far been performed on tear fluid from patients with SS [22, 23], we were interested in analysing tear fluid collected from patients with pSS and healthy controls, in combination with whole saliva from the same individuals, in order to explore novel biomarkers encompassing both lacrimal and salivary disease target organs. We identified overexpression of proteins involved in TNF-α signalling (CPNE1) and B cell survival (PRDX3), in the Krebs cycle (ACO2) and in oxidative stress (APEX1) in tear fluid from patients with pSS (Table 5, Additional file 4: Figure S3). Moreover, two protein-protein interaction networks encompassing the upregulated proteins in tear fluid from patients with pSS were visualised using STRING. These networks were involved in oxidative stress, and the formation of the cytoskeleton and cell migration, respectively. With a vital role in regulating NF-κ B activation, and in turn B cell survival, PRDX3 was present within the protein network involving redox reactions and oxidative stress (Fig. 4).

Additionally, we explored protein expression in whole saliva and tear fluid simultaneously, and out of all the aforementioned proteins in the study, LCN2 was found to be upregulated in both fluids in the patients with pSS. Hence, the most upregulated protein in whole saliva from patients with pSS identified in our current analysis (Table 3), is also a key player in tear fluid from these same individuals. Being an iron-binding protein involved in the innate immune system and the activation of neutrophils [50], LCN2 could be viewed as a possible biomarker for SS, whereby screening for LCN2 in whole saliva and tear fluid from patients with pSS could provide additional diagnostic accuracy. This observation further strengthens the notion of a role for viral infection in the pathogenesis of SS. Interestingly, LCN2 has also been previously proposed as a potential disease biomarker in the autoimmune disease systemic lupus erythematosus (SLE), as elevated levels of anti-LCN2 were detected in serum samples from patients with SLE [54].

In spite of low tear fluid volumes collected individually from each patient with pSS, leading to few attainable data from the EVs isolated individually from each participant, our proteomic analysis of EVs extracted from the pooled tear sample combined from 11 patients with pSS revealed that CPNE1 and CALM were expressed more in the patient group. The presence of both these upregulated proteins indicates how their cellular functions fulfil each other, with CPNE1 playing a central role in inflammation and apoptosis due to TNF-α signalling, while CALM is essential in cell signaling and the activation of the immune system.

Conclusions

In conclusion, the application of LC-MS alone and in combination with size-exclusion chromatography, to both stimulated whole saliva and tear fluid from patients with pSS, allowed the isolation of EVs and the screening for novel biomarkers encompassing both salivary and lacrimal disease target organs. These biomarkers include LCN2, APMAP and CPNE1. The screening for such biomarkers in whole saliva and tear fluid from patients with pSS as a part of the diagnostic process could provide additional diagnostic accuracy. Furthermore, saliva and tear fluid represent attractive mediums for diagnosis using proteomic analysis, as collection of these samples is not invasive, their composition is not complex and the analysis may be easily repeated for monitoring the disease over time. As a next step, the validation of these biomarkers in larger SS cohorts is needed, whereby disease stratification can be explored in relation to protein expression levels. It is also of interest to validate these biomarkers in related diseases such as sicca syndrome and secondary SS. By doing so, one can explore whether these biomarkers can also be applied to monitor disease progression, and thereafter explore more strategic targeted therapeutic approaches in SS.

Additional files

Additional file 1: Table S1. Upregulated proteins in whole saliva from patients with pSS. **Table S2.** Upregulated proteins in EVs isolated from whole saliva from patients with pSS. **Table S3.** Upregulated proteins in tear fluid from patients with pSS (DOC 348 kb)

Additional file 2: Figure S1. Spectral count representation of highly upregulated pSS-associated proteins identified in stimulated whole saliva. Spectral counts from individual stimulated whole saliva samples from patients with pSS (*black circles*) and controls (*white circles*) showing little or none of the proteins LCN2, GRN, CALM, NPC2 and CALML5 in controls compared to patients with pSS (TIF 8483 kb)

Additional file 3: Figure S2. Spectral count representation of highly upregulated pSS-associated proteins detected in EVs from whole saliva. Spectral counts from individual EV samples isolated from whole saliva from patients with pSS (*black circles*) and controls (*white circles*) showing little or none of the proteins APMAP, GNA13, WDR1, SIRPA and LSP1 in controls compared to patients with pSS (TIF 8483 kb)

Additional file 4: Figure S3. Spectral count representation of highly upregulated pSS-associated proteins identified in tear fluid. Spectral counts from individual tear samples of patients with pSS (*black circles*) and controls (*white circles*) showing little or none of the proteins APEX1, PRDX3, CPNE1, ACO2 and LMO7 in controls compared to patients with pSS (TIF 8483 kb)

Abbreviations

ACO2: Aconitate hydratase; AECG: American-European Consensus Group; APEX1: DNA-(apurinic or apyrimidinic site) lyase; APMAP: Adipocyte plasma membrane-associated protein; BSA: Bovine serum albumin; CALM: Calmodulin; CALML5: Calmodulin-like protein 5; CPNE1: Copine; DAVID: Database for Annotation, Visualization and Integrated Discovery; ELISA: enzyme-linked immunosorbent assay; ESP1: Epididymal secretory protein 1; EV: Extracellular vesicles; FDR: False discovery rate; GNA13: Guanine nucleotide-binding protein subunit alpha-13; GO: Gene Ontology; GRN: Granulins; IL: Interleukin; LC-MS: Liquid chromatography-mass spectrometry; LCN2: Neutrophil gelatinase-associated lipocalin; LMO7: LIM domain only protein 7; LSP1: Lymphocyte-specific protein 1; MFI: Median fluorescence intensity; PBS: phosphate-buffered saline; NK: Natural killer; PRDX3: Thioredoxin-dependent peroxidase reductase; pSS: primary Sjögren's syndrome; S/N: Signal-to-noise ratio; SIRPA: Tyrosine-protein phosphatase non-receptor type substrate 1; SLE: Systemic lupus erythematosus; SS: Sjögren's syndrome; STRING: Search Tool for the Retrieval of Interacting Genes/Proteins; TIC: Total ion current; WDR1: WD repeat-containing protein 1

Acknowledgements

We express our gratitude to the patients and controls who took part in our study. Their collaboration has been essential for the accomplishment of the study presented. We appreciatively acknowledge Ann-Kristin Molværsmyr and Ann-Kristin Ruus for excellent technical assistance. We further express our sincere appreciation to Dr Sten Ræder, the head of the Norwegian Dry Eye Clinic; Dr Benedikte Døskeland from the Rheumatology Department at Rikshospitalet; and all other staff members at the research and clinical institutions involved for their effort and dedication.

Funding

The study was supported by the Faculty of Dentistry at the University of Oslo.

Availability of data and materials

The datasets generated and/or analysed during the current study are not publicly available due to ethical restrictions enforced by the research and medical institutions under license for the current study. Data are, however, available from the authors upon reasonable request and with permission of the Regional Medical Ethical Committee of South-East Norway, the University of Oslo and Oslo University Hospital.

Authors' contributions

LA designed the study, recruited the patients and controls, collected the clinical data, carried out the laboratory experiments, examined and interpreted the proteomics and EV data, performed statistical analyses and drafted and critically revised the manuscript. HKG designed and supervised the study, examined and interpreted the proteomics and EV data, performed statistical analyses and drafted and critically revised the manuscript. BV designed the study, carried out the laboratory experiments, examined and interpreted the EV data, performed statistical analyses and critically revised the manuscript. RØ designed the study, carried out the laboratory experiments, examined and interpreted the EV data, performed statistical analyses and critically revised the manuscript. BT carried out the laboratory experiments, examined and interpreted the proteomics data, performed statistical analyses and revised the manuscript. SR recruited the patients and controls, collected the clinical data and performed the laboratory experiments. AY designed the study, collected the clinical data and critically revised the manuscript. EG performed the laboratory experiments and critically revised the manuscript. TPU designed the study and critically revised the manuscript. XC collected the clinical data and critically revised the manuscript. ØAU collected the clinical data and critically revised the manuscript. ØP recruited the patients, collected the clinical data and critically revised the manuscript. JLJ designed and supervised the study, recruited the patients and controls, collected the clinical data and critically revised the manuscript. All authors read and approved the final manuscript.

Competing interests

The authors declare that they have no competing interests.

Consent for publication

Consent for publication has been obtained from all participants.

Ethics approval and consent to participate

This study was approved by the Regional Medical Ethical Committee of South-East Norway (2015/363). Moreover, all studied subjects gave their informed written consent to participate in the study.

Author details

[1]Department of Oral Surgery and Oral Medicine, Faculty of Dentistry, University of Oslo, Oslo, Norway. [2]Department of Oral Biology, University of Oslo, Oslo, Norway. [3]Department of Medical Biochemistry, Oslo University Hospital, Oslo, Norway. [4]Department of Biosciences, University of Oslo, Oslo, Norway. [5]Department of Cariology and Gerodontology, University of Oslo, Oslo, Norway. [6]The Norwegian Dry Eye Clinic, Oslo, Norway. [7]Department of Ophthalmology, Oslo University Hospital, Oslo, Norway. [8]Department of Rheumatology, Oslo University Hospital, Oslo, Norway.

Received: 25 October 2016 Accepted: 12 January 2017
Published online: 25 January 2017

References

1. Jonsson R, Bolstad AI, Brokstad KA, Brun JG. Sjogren's syndrome–a plethora of clinical and immunological phenotypes with a complex genetic background. Ann NY Acad Sci. 2007;1108:433–47.
2. Jonsson R, Vogelsang P, Volchenkov R, Espinosa A, Wahren-Herlenius M, Appel S. The complexity of Sjogren's syndrome: novel aspects on pathogenesis. Immunol Lett. 2011;141:1–9.
3. Ramos-Casals M, Brito-Zeron P, Siso-Almirall A, Bosch X, Tzioufas AG. Topical and systemic medications for the treatment of primary Sjogren's syndrome. Nat Rev Rheumatol. 2012;8:399–411.
4. Bowman SJ, Ibrahim GH, Holmes G, Hamburger J, Ainsworth JR. Estimating the prevalence among Caucasian women of primary Sjogren's syndrome in two general practices in Birmingham, UK. Scand J Rheumatol. 2004;33:39–43.
5. Goransson LG, Haldorsen K, Brun JG, Harboe E, Jonsson MV, Skarstein K, Time K, Omdal R. The point prevalence of clinically relevant primary Sjogren's syndrome in two Norwegian counties. Scand J Rheumatol. 2011;40:221–4.
6. Maldini C, Seror R, Fain O, Dhote R, Amoura Z, De Bandt M, Delassus JL, Falgarone G, Guillevin L, Le Guern V, et al. Epidemiology of primary Sjogren's syndrome in a French multiracial/multiethnic area. Arthritis Care Res (Hoboken). 2014;66:454–63.

7. Vitali C, Bombardieri S, Jonsson R, Moutsopoulos HM, Alexander EL, Carsons SE, Daniels TE, Fox PC, Fox RI, Kassan SS, et al. Classification criteria for Sjogren's syndrome: a revised version of the European criteria proposed by the American-European Consensus Group. Ann Rheum Dis. 2002;61:554–8.
8. Jonsson R, Theander E, Sjostrom B, Brokstad K, Henriksson G. Autoantibodies present before symptom onset in primary Sjogren syndrome. JAMA. 2013; 310:1854–5.
9. Chisholm DM, Mason DK. Labial salivary gland biopsy in Sjogren's disease. J Clin Pathol. 1968;21:656–60.
10. Daniels TE, Whitcher JP. Association of patterns of labial salivary gland inflammation with keratoconjunctivitis sicca. Analysis of 618 patients with suspected Sjogren's syndrome. Arthritis Rheum. 1994;37:869–77.
11. Giovelli RA, Santos MC, Serrano EV, Valim V. Clinical characteristics and biopsy accuracy in suspected cases of Sjogren's syndrome referred to labial salivary gland biopsy. BMC Musculoskelet Disord. 2015;16:30.
12. Baldini C, Giusti L, Bazzichi L, Lucacchini A, Bombardieri S. Proteomic analysis of the saliva: a clue for understanding primary from secondary Sjogren's syndrome? Autoimmun Rev. 2008;7:185–91.
13. Giusti L, Baldini C, Bazzichi L, Bombardieri S, Lucacchini A. Proteomic diagnosis of Sjogren's syndrome. Expert Rev Proteomics. 2007;4:757–67.
14. Giusti L, Baldini C, Bazzichi L, Ciregia F, Tonazzini I, Mascia G, Giannaccini G, Bombardieri S, Lucacchini A. Proteome analysis of whole saliva: a new tool for rheumatic diseases – the example of Sjogren's syndrome. Proteomics. 2007;7:1634–43.
15. Giusti L, Baldini C, Ciregia F, Giannaccini G, Giacomelli C, De Feo F, Delle Sedie A, Riente L, Lucacchini A, Bazzichi L, Bombardieri S. Is GRP78/BiP a potential salivary biomarker in patients with rheumatoid arthritis? Proteomics Clin Appl. 2010;4:315–24.
16. Ryu OH, Atkinson JC, Hoehn GT, Illei GG, Hart TC. Identification of parotid salivary biomarkers in Sjogren's syndrome by surface-enhanced laser desorption/ionization time-of-flight mass spectrometry and two-dimensional difference gel electrophoresis. Rheumatology (Oxford). 2006;45:1077–86.
17. Peluso G, De Santis M, Inzitari R, Fanali C, Cabras T, Messana I, Castagnola M, Ferraccioli GF. Proteomic study of salivary peptides and proteins in patients with Sjogren's syndrome before and after pilocarpine treatment. Arthritis Rheum. 2007;56:2216–22.
18. Hu S, Wang J, Meijer J, Ieong S, Xie Y, Yu T, Zhou H, Henry S, Vissink A, Pijpe J, et al. Salivary proteomic and genomic biomarkers for primary Sjogren's syndrome. Arthritis Rheum. 2007;56:3588–600.
19. Fleissig Y, Deutsch O, Reichenberg E, Redlich M, Zaks B, Palmon A, Aframian DJ. Different proteomic protein patterns in saliva of Sjogren's syndrome patients. Oral Dis. 2009;15:61–8.
20. Hu S, Vissink A, Arellano M, Roozendaal C, Zhou H, Kallenberg CG, Wong DT. Identification of autoantibody biomarkers for primary Sjogren's syndrome using protein microarrays. Proteomics. 2011;11:1499–507.
21. Delaleu N, Mydel P, Kwee I, Brun JG, Jonsson MV, Jonsson R. High fidelity between saliva proteomics and the biologic state of salivary glands defines biomarker signatures for primary Sjogren's syndrome. Arthritis Rheumatol. 2015;67:1084–95.
22. Tomosugi N, Kitagawa K, Takahashi N, Sugai S, Ishikawa I. Diagnostic potential of tear proteomic patterns in Sjogren's syndrome. J Proteome Res. 2005;4:820–5.
23. Li B, Sheng M, Li J, Yan G, Lin A, Li M, Wang W, Chen Y. Tear proteomic analysis of Sjogren syndrome patients with dry eye syndrome by two-dimensional-nano-liquid chromatography coupled with tandem mass spectrometry. Sci Rep. 2014;4:5772.
24. Yoshizawa JM, Schafer CA, Schafer JJ, Farrell JJ, Paster BJ, Wong DT. Salivary biomarkers: toward future clinical and diagnostic utilities. Clin Microbiol Rev. 2013;26:781–91.
25. Baldini C, Gallo A, Perez P, Mosca M, Alevizos I, Bombardieri S. Saliva as an ideal milieu for emerging diagnostic approaches in primary Sjogren's syndrome. Clin Exp Rheumatol. 2012;30:785–90.
26. Denny P, Hagen FK, Hardt M, Liao L, Yan W, Arellanno M, Bassilian S, Bedi GS, Boontheung P, Cociorva D, et al. The proteomes of human parotid and submandibular/sublingual gland salivas collected as the ductal secretions. J Proteome Res. 2008;7:1994–2006.
27. Altelaar AF, Munoz J, Heck AJ. Next-generation proteomics: towards an integrative view of proteome dynamics. Nat Rev Genet. 2013;14:35–48.
28. Baldini C, Giusti L, Ciregia F, Da Valle Y, Giacomelli C, Donadio E, Sernissi F, Bazzichi L, Giannaccini G, Bombardieri S, Lucacchini A. Proteomic analysis of saliva: a unique tool to distinguish primary Sjogren's syndrome from secondary Sjogren's syndrome and other sicca syndromes. Arthritis Res Ther. 2011;13:R194.
29. Ambatipudi KS, Swatkoski S, Moresco JJ, Tu PG, Coca A, Anolik JH, Gucek M, Sanz I, Yates 3rd JR, Melvin JE. Quantitative proteomics of parotid saliva in primary Sjogren's syndrome. Proteomics. 2012;12:3113–20.
30. Iwai K, Minamisawa T, Suga K, Yajima Y, Shiba K. Isolation of human salivary extracellular vesicles by iodixanol density gradient ultracentrifugation and their characterizations. J Extracell Vesicles. 2016;5:30829.
31. Yanez-Mo M, Siljander PR, Andreu Z, Zavec AB, Borras FE, Buzas EI, Buzas K, Casal E, Cappello F, Carvalho J, et al. Biological properties of extracellular vesicles and their physiological functions. J Extracell Vesicles. 2015;4:27066.
32. Iraci N, Leonardi T, Gessler F, Vega B, Pluchino S. Focus on extracellular vesicles: physiological role and signalling properties of extracellular membrane vesicles. Int J Mol Sci. 2016;17:171.
33. Raposo G, Stoorvogel W. Extracellular vesicles: exosomes, microvesicles, and friends. J Cell Biol. 2013;200:373–83.
34. Wang JG, Williams JC, Davis BK, Jacobson K, Doerschuk CM, Ting JP, Mackman N. Monocytic microparticles activate endothelial cells in an IL-1beta-dependent manner. Blood. 2011;118:2366–74.
35. Oehmcke S, Morgelin M, Malmstrom J, Linder A, Chew M, Thorlacius H, Herwald H. Stimulation of blood mononuclear cells with bacterial virulence factors leads to the release of pro-coagulant and pro-inflammatory microparticles. Cell Microbiol. 2012;14:107–19.
36. Nielsen CT, Ostergaard O, Stener L, Iversen LV, Truedsson L, Gullstrand B, Jacobsen S, Heegaard NH. Increased IgG on cell-derived plasma microparticles in systemic lupus erythematosus is associated with autoantibodies and complement activation. Arthritis Rheum. 2012;64:1227–36.
37. van Eijk IC, Tushuizen ME, Sturk A, Dijkmans BA, Boers M, Voskuyl AE, Diamant M, Wolbink GJ, Nieuwland R, Nurmohamed MT. Circulating microparticles remain associated with complement activation despite intensive anti-inflammatory therapy in early rheumatoid arthritis. Ann Rheum Dis. 2010;69:1378–82.
38. Ismail N, Wang Y, Dakhlallah D, Moldovan L, Agarwal K, Batte K, Shah P, Wisler J, Eubank TD, Tridandapani S, et al. Macrophage microvesicles induce macrophage differentiation and miR-223 transfer. Blood. 2013;121:984–95.
39. Eken C, Gasser O, Zenhaeusern G, Oehri I, Hess C, Schifferli JA. Polymorphonuclear neutrophil-derived ectosomes interfere with the maturation of monocyte-derived dendritic cells. J Immunol. 2008;180:817–24.
40. Lugini L, Cecchetti S, Huber V, Luciani F, Macchia G, Spadaro F, Paris L, Abalsamo L, Colone M, Molinari A, et al. Immune surveillance properties of human NK cell-derived exosomes. J Immunol. 2012;189:2833–42.
41. MacKenzie A, Wilson HL, Kiss-Toth E, Dower SK, North RA, Surprenant A. Rapid secretion of interleukin-1beta by microvesicle shedding. Immunity. 2001;15:825–35.
42. Zhang HG, Liu C, Su K, Yu S, Zhang L, Zhang S, Wang J, Cao X, Grizzle W, Kimberly RP. A membrane form of TNF-alpha presented by exosomes delays T cell activation-induced cell death. J Immunol. 2006;176:7385–93.
43. Mathivanan S, Lim JW, Tauro BJ, Ji H, Moritz RL, Simpson RJ. Proteomics analysis of A33 immunoaffinity-purified exosomes released from the human colon tumor cell line LIM1215 reveals a tissue-specific protein signature. Mol Cell Proteomics. 2010;9:197–208.
44. Böing AN, van der Pol E, Grootemaat AE, Coumans FAW, Sturk A, Nieuwland R. Single-step isolation of extracellular vesicles by size-exclusion chromatography. J Extracellular Vesicles. 2014;3:10.3402/jev.v3.23430. doi:10.3402/jev.v3.23430.
45. Sun Y, Xia Z, Shang Z, Sun K, Niu X, Qian L, Fan LY, Cao CX, Xiao H. Facile preparation of salivary extracellular vesicles for cancer proteomics. Sci Rep. 2016;6:24669.
46. Thery C, Regnault A, Garin J, Wolfers J, Zitvogel L, Ricciardi-Castagnoli P, Raposo G, Amigorena S. Molecular characterization of dendritic cell-derived exosomes. Selective accumulation of the heat shock protein hsc73. J Cell Biol. 1999;147:599–610.
47. Vlassov AV, Magdaleno S, Setterquist R, Conrad R. Exosomes: current knowledge of their composition, biological functions, and diagnostic and therapeutic potentials. Biochim Biophys Acta. 2012;1820:940–8.
48. Kowal J, Arras G, Colombo M, Jouve M, Morath JP, Primdal-Bengtson B, Dingli F, Loew D, Tkach M, Thery C. Proteomic comparison defines novel markers to characterize heterogeneous populations of extracellular vesicle subtypes. Proc Natl Acad Sci USA. 2016;113:E968–77.
49. Ostergaard O, Nielsen CT, Iversen LV, Jacobsen S, Tanassi JT, Heegaard NH. Quantitative proteome profiling of normal human circulating microparticles. J Proteome Res. 2012;11:2154–63.

50. Pawar RD, Goilav B, Xia Y, Zhuang H, Herlitz L, Reeves WH, Putterman C. Serum autoantibodies in pristane induced lupus are regulated by neutrophil gelatinase associated lipocalin. Clin Immunol. 2014;154:49–65.
51. Thurner L, Preuss KD, Fadle N, Regitz E, Klemm P, Zaks M, Kemele M, Hasenfus A, Csernok E, Gross WL, et al. Progranulin antibodies in autoimmune diseases. J Autoimmun. 2013;42:29–38.
52. Sun BK, Boxer LD, Ransohoff JD, Siprashvili Z, Qu K, Lopez-Pajares V, Hollmig ST, Khavari PA. CALML5 is a ZNF750- and TINCR-induced protein that binds stratifin to regulate epidermal differentiation. Genes Dev. 2015;29:2225–30.
53. Skarstein K, Aqrawi LA, Oijordsbakken G, Jonsson R, Jensen JL. Adipose tissue is prominent in salivary glands of Sjogren's syndrome patients and appears to influence the microenvironment in these organs. Autoimmunity. 2016;49:338–46.
54. Li YN, Hu FL, Dai YJ, Li R, Ma XX, Du Y, Feng M, Jia Y, Zhang CF, Zhu L, et al. Serum anti-lipocalin 2 IgG is a novel biomarker in the diagnosis of systemic lupus erythematosus. Lupus. 2014;23:868–75.

Submit your next manuscript to BioMed Central and we will help you at every step:

- We accept pre-submission inquiries
- Our selector tool helps you to find the most relevant journal
- We provide round the clock customer support
- Convenient online submission
- Thorough peer review
- Inclusion in PubMed and all major indexing services
- Maximum visibility for your research

Submit your manuscript at
www.biomedcentral.com/submit

BioMed Central
